# Accelerated biological aging and risk of depression and anxiety: evidence from 424,299 UK Biobank participants

**DOI:** 10.1038/s41467-023-38013-7

**Published:** 2023-04-20

**Authors:** Xu Gao, Tong Geng, Meijie Jiang, Ninghao Huang, Yinan Zheng, Daniel W. Belsky, Tao Huang

**Affiliations:** 1 Department of Occupational and Environmental Health Sciences, School of Public Health, Peking University, Beijing, China; 2 Peking University Sixth Hospital, Peking University Institute of Mental Health, NHC Key Laboratory of Mental Health (Peking University), National Clinical Research Center for Mental Disorders (Peking University Sixth Hospital), Beijing, China; 3 Department of Epidemiology and Biostatistics, School of Public Health, Peking University, Beijing, China; 4 Department of Preventive Medicine, Feinberg School of Medicine, Northwestern University, Chicago, IL USA; 5 Department of Epidemiology & Butler Columbia Aging Center, Columbia University, New York, NY USA

**Keywords:** Epidemiology, Depression, Predictive markers, Anxiety

## Abstract

Theory predicts that biological processes of aging may contribute to poor mental health in late life. To test this hypothesis, we evaluated prospective associations between biological age and incident depression and anxiety in 424,299 UK Biobank participants. We measured biological age from clinical traits using the KDM-BA and PhenoAge algorithms. At baseline, participants who were biologically older more often experienced depression/anxiety. During a median of 8.7 years of follow-up, participants with older biological age were at increased risk of incident depression/anxiety (5.9% increase per standard deviation [SD] of KDM-BA acceleration, 95% confidence intervals [CI]: 3.3%–8.5%; 11.3% increase per SD of PhenoAge acceleration, 95% CI: 9.%–13.0%). Biological-aging-associated risk of depression/anxiety was independent of and additive to genetic risk measured by genome-wide-association-study-based polygenic scores. Advanced biological aging may represent a potential risk factor for incident depression/anxiety in midlife and older adults and a potential target for risk assessment and intervention.

## Abstract (web summary)

Theory indicates that biological aging may contribute to poor mental health in late life. Here, authors show advanced biological aging may represent a potential risk factor for incident depression/anxiety in midlife and older adults and a potential target for risk assessment and intervention.

## Introduction

Depression and anxiety are common mental disorders that often co-occur and are associated with increased disability and mortality, especially in older adults^1^. Prevention of depression and anxiety in older adults therefore has potential to mitigate disease burden in an aging population^2^. Identification of risk factors and mechanisms of vulnerability to mental disorders is a public health priority. Recent reports from multiple cohorts reveal poor mental health as a risk factor for more advanced and faster biological aging, including self-reported unsuccessful aging^3^, accelerated brain aging^4^, shorter leukocyte telomere length^5^, epigenetic aging measured from blood DNA methylation profiles^6^, and older biological age and faster pace of aging as measured from blood chemistries and other clinical traits^7^. However, nearly all work to date had focused on the hypothesis that poor mental health accelerates processes of biological aging^8^. A complementary, but less-studied hypothesis is that accelerated processes of biological aging may, themselves, pose risks to depression/anxiety disorders of older adults^9^.

Aging is a complex biological process that progressively undermines the integrity and resilience capacity of cells, tissues, and organs^10^. Ideal measurements of biological aging should thus reflect the landscape of aging in multiple biological systems^11^. Several measurements of biological aging have been proposed, ranging from individual biomarkers such as telomere length to algorithms that integrate information across epigenetic, proteomic, metabolomic, and other molecular levels of analysis^12,13^. Among these, algorithms that combine information from standard clinical parameters have proven to be among the most accurate for predicting morbidity and mortality^14,15^. To test the hypothesis that accelerated biological aging could increase the risk for depression/anxiety, we applied two published and validated clinical-parameter biological-age algorithms to blood chemistries collected from ~0.4 million UK Biobank participants at their baseline assessment and linked the computed biological age data with health records compiled over ~nine years of follow-up. We computed biological age values for participants using the Klemera-Doubal method Biological Age (KDM-BA) and the PhenoAge algorithms^16,17^, both of which have been validated in multi-ethnic cohorts of older adults to predict disease, disability, and mortality^18,19^. KDM-BA models biological age as the average biological state associated with a particular chronological age in a reference population. This model assumes biological age increases linearly with time. PhenoAge models biological age as the average biological state associated with a particular level of mortality risk in a reference population. This model assumes biological age increases exponentially with time. Following our approach in past projects, we used both algorithms to establish the robustness of findings to assumptions about the correct way to model biological aging^20,21^.

In this work, we tested the associations of the two biological ages with the risks of depression/anxiety in the UK Biobank cohort. The study shows that advanced biological aging could be a potential risk factor for incident depression/anxiety in midlife and older adults and a potential target for risk assessment and intervention. Our analysis proceeded in three steps (Figure S1). First, we tested if participants who were biologically older at baseline were more likely to have prevalent depression or anxiety as compared to peers of the same chronological age who were measured to be biologically younger. This analysis included in the full set of UK Biobank participants with available data on biological age and depression/anxiety at baseline (N = 424,299). Second, we tested if participants who were biologically older at baseline were more likely to have incident depression/anxiety over follow-up as compared to peers of the same chronological age who were measured to be biologically younger. This analysis included the subset of participants who were free of depression and anxiety at baseline and who were followed-up through healthcare records and a follow-up survey (N = 369,745). Third, we repeated this second analysis among the subset of participants free of depression and anxiety at baseline who completed the follow-up survey (N = 124,976). The purpose of this third analysis was to evaluate the extent to which reliance on healthcare records might bias association results. We additionally conducted a secondary analysis of UK Biobank genetic data to evaluate the potential gene-environment interplay between genetic risk for depression and anxiety and the internal physiological environment reflected by participants’ biological aging status.

## Results

### Participants’ characteristics and biological ages

We analyzed data for three overlapping groups of participants. The first group included all individuals providing baseline blood chemistry data required for calculation of biological age measurements and who completed mental health surveys at enrollment baseline (N = 424,299). The second group consisted of the subset of the first group who did not have prevalent depression/anxiety at baseline (N = 369,745). The third group was the subset of individuals who did not have prevalent depression/anxiety at baseline and who also participated in the online follow-up mental health survey (N = 124,976). Characteristics of these three groups are reported in Table 1. Briefly, the participants were mostly White (>95%), 46.2% were male, aged 57 ± 8-year-old, with a substantial prevalence of hypertension (54%) and some prevalence of coronary heart disease (3–5%) and diabetes (3–5%). In the first group, baseline PHQ-4 scores averaged 1.58 ± 2.08. Based on PHQ-4 scores and hospital records, 13% met the criteria for having symptoms of depression (6%) and anxiety (10%). In the second group, who were free of depression and anxiety at baseline, depression, and anxiety were incident in 4.47% (N_depression_ = 11,402, N_anxiety_ = 8,472, and N_both_ = 2,991) over a median of 8.7 years of follow-up. In the third group, who were free of depression/anxiety at baseline and who participated in the follow-up survey, depression, and anxiety were incident in 4.9% (N_depression_= 4,230, N_anxiety_ = 3,347, and N_both_ = 1,501) at online follow-up (median follow-up = 8.7 years).

**Table 1:** Characteristics of study participants^a^

Characteristic	Analysis 1 (*N* = 424,299)	Analysis 2 (*N* = 369,745)	Analysis 3 (N = 124,976)
**Age (years)**	56.47 (8.07)	56.75 (8.04)	56.07 (7.73)
**Body mass index (BMI, kg/m**^**2**^**)**			
Underweight or normal weight (<25)	142453 (33.6%)	126675 (34.3%)	49405 (39.5%)
Overweight (25 to <30)	181232 (42.7%)	160278 (43.3%)	52451 (42.0%)
Obese (≥30)	100614 (23.7%)	82792 (22.4%)	23120 (18.5%)
**Sex (male)**	196105 (46.2%)	173971 (47.0%)	56178 (44.9%)
**Race (white)**	405882 (95.7%)	355974 (96.3%)	121908 (97.5%)
**Smoking status**			
Current smoker	43230 (10.2%)	34109 (9.2%)	8366 (6.7%)
Former smoker	148600 (35.0%)	130917 (35.4%)	44220 (35.4%)
Never smoker	232469 (54.8%)	204719 (55.4%)	72390 (57.9%)
**Healthy alcohol intake (yes)** ^**b**^	210583 (49.6%)	187931 (50.8%)	67205 (53.8%)
**Healthy physical activity (yes)**^**c**^	302987 (71.4%)	269590 (72.9%)	92114 (73.7%)
**Townsend deprivation index**^**d**^	−1.43 (3.02)	−1.57 (2.93)	−1.81 (2.77)
**Major diseases**			
Hypertension^e^	229252 (54.0%)	201136 (54.4%)	62896 (50.3%)
Coronary heart disease^f^	23274 (5.5%)	18335 (5.0%)	4121 (3.3%)
Diabetes^g^	20849 (4.9%)	16506 (4.5%)	3819 (3.1%)
**Biological ages**			
KDM-BA	48.76 (12.18)	48.74 (12.24)	47.42 (11.88)
KDM-BA acceleration	−7.71 (9.38)	−8.00 (9.41)	−8.65 (9.54)
PhenoAge	45.55 (9.99)	45.63 (9.90)	44.37 (9.36)
PhenoAge acceleration	−10.92 (5.45)	−11.11 (5.27)	−11.70 (4.95)
**Components of biological ages**			
FEV_1_ (L)^*^	2.83 (0.76)	2.85 (0.76)	2.92 (0.75)
SBP (mm Hg)^*^	139.64 (19.61)	140.09 (19.63)	138.48 (19.12)
Total Cholesterol (mg/dL)^*^	220.34 (43.04)	220.82 (42.82)	221.80 (41.69)
Glycated hemoglobin (%)^*^	5.44 (0.58)	5.43 (0.56)	5.37 (0.48)
Blood urea nitrogen (mg/dL)^*^	15.12 (3.77)	15.16 (3.70)	14.95 (3.50)
Lymphocyte (%)^#^	28.90 (7.33)	28.93 (7.29)	29.13 (7.19)
Mean cell volume (fL)^#^	82.86 (5.16)	82.86 (5.10)	82.84 (4.97)
Serum glucose (mg/dL)^#^	91.71 (20.53)	91.50 (19.60)	90.63 (17.29)
Red cell distribution width (%)^#^	13.48 (0.95)	13.46 (0.92)	13.42 (0.89)
White blood cell count (1000 cells/uL)^#^	6.85 (1.91)	6.81 (1.88)	6.65 (1.76)
Albumin (g/dL)^*#^	4.52 (0.24)	4.52 (0.24)	4.54 (0.24)
Creatinine (mg/dL)^*#^	0.82 (0.18)	0.82 (0.18)	0.81 (0.16)
C-reactive protein (mg/dL)^*#^	0.25 (0.42)	0.24 (0.40)	0.21 (0.37)
Alkaline phosphatase (U/L)^*#^	83.06 (25.60)	82.64 (25.20)	80.64 (23.24)
**Mental health status at baseline**			
PHQ-4 score	1.58 (2.08)	/	/
Depression/anxiety disorders	54554 (12.9%)	/	/
Depression	26424 (6.2%)	/	/
Anxiety	43544 (10.3%)	/	/
**Mental health status at follow-up survey**			
PHQ-9 score	/	/	2.27 (3.05)
GAD-7 score	/	/	1.75 (2.89)
Depression/anxiety disorders	/	/	6076 (4.9%)
Depression	/	/	4230 (3.4%)
Anxiety	/	/	3347 (2.7%)

^a^Included the all 424,299 participants for Analysis 1, the subgroup of 369,745 participants that were free of depression/anxiety at baseline for Analysis 2, and the subgroup of 124,976 participants that were free of depression/anxiety at baseline and with follow-up survey data for Analysis 3; Mean values (standard deviation) for continuous variables and n (%) for categorical variables.

^b^Healthy alcohol intake: male: <28 g/day; female: <14 g/day.

^c^Healthy physical activity: ≥150 min/week moderate or ≥75 min/week vigorous or 150 min/week mixed (moderate + vigorous) activity.

^d^This index is composite score based on four key variables: unemployment, overcrowded household, non–car ownership, and non–home ownership.

^e^Hypertension diagnosed by doctor.

^f^Coronary heart disease diagnosed by doctor.

^g^Diabetes diagnosed by doctor.

^*^Employed to construct KDM-BA.

^#^Employed to construct PhenoAge.

At baseline, participants’ biological ages were highly correlated with their chronological ages. After residualizing measures for chronological age, the residual values of KDM-BA and PhenoAge (referred to as “age acceleration; AA”) remained correlated (Pearson coefficient = 0.23; Fig. S2)

### Biological aging and prevalence of depression/anxiety at baseline

**Fig. 1:**
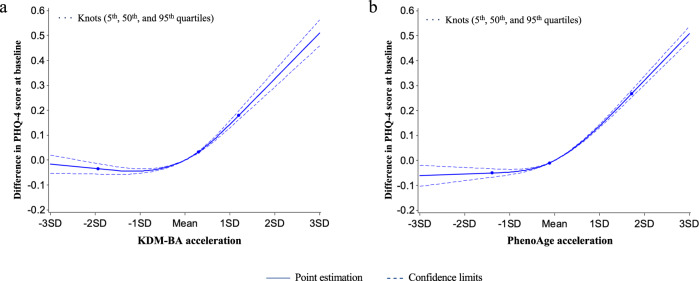
Graphs of the best fitting models for relationships of KDM-BA acceleration and PhenoAge acceleration with PHQ-4 score at baseline. Panels: KDM-BA acceleration (**a**) and PhenoAge acceleration (**b**). Solid line: Point estimation; Dash line: Confidence limits; Dots: Knots (5th, 50th, and 95th percentiles). Restricted cubic spline regression model adjusted for age, sex, BMI category, race, smoking status (current/former/never), healthy alcohol intake status, healthy physical activity status, Townsend deprivation index, and prevalent hypertension, CHD, and diabetes. Source data are provided as a Source Data file.

Participants with older biological age had higher PHQ-4 scores and were more likely to have a diagnosis of depression or anxiety at baseline as compared with peers of the same chronological age who were biologically younger (Table S1). Covariate adjustment for socioeconomic factors, health behaviors, and prevalent chronic diseases attenuated effect-size estimates. However, most associations remained statistically significant (*p*-values <0.05; Table 2); in the fully-adjustment model, participants with KDM-BA values one-standard deviation (SD) older the expectation for their chronological age scored 0.0516-units (Standard error = 0.0045) higher on the PHQ-4, and had 12.3% higher odds of depression/anxiety (95% confidence intervals [CI]: 10.8%–13.8%; for depression, 9.6%, 95% CI: 7.6–11.7%; for anxiety, 13.5%, 95% CI: 11.8%–15.2%; all *p*-values <0.0001). Results were similar for the PhenoAge, with somewhat larger effect sizes (Table 2). For both KDM-BA and PhenoAge, associations with PHQ-4 scores and disorders were monotonic and non-linear across quartiles of biological age (Table 2 and Fig. 1). For instance, compared to Q1 of KDM-BA acceleration, the odds ratios (ORs) of either disorder were 1.135 (95% CI: 1.100–1.170), 1.199 (95% CI: 1.156–1.243), and 1.325 (95% CI: 1.276–1.375), respectively.

**Table 2:** Associations of the biological age accelerations with the PHQ-4 Score and odds of depression/anxiety disorders at baseline (fully adjusted model)^a^

Biological age	PHQ-4 Score		Depression/anxiety disorders			Depression			Anxiety		
	Coefficients (SE)	*p*-value	N_case_/N_total_	Odds ratio (95% CI)	*p*-value	N_case_/N_total_	Odds ratio (95% CI)	*p*-value	N_case_/N_total_	Odds ratio (95% CI)	*p*-value
KDM-BA acceleration (Continuous)	0.0516 (0.0045)	**<0.0001**	54554/424299	1.123 (1.108 – 1.138)	**<0.0001**	26424/424299	1.096 (1.076 – 1.117)	**<0.0001**	43544/424299	1.135 (1.118 – 1.152)	**<0.0001**
**KDM-BA acceleration (Quartiles)**											
Q1	Ref		9839/106074	Ref		4921/106074	Ref		7555/106074	Ref	
Q2	0.0405 (0.0096)	**<0.0001**	12841/106075	1.135 (1.100 – 1.170)	**<0.0001**	6191/106075	1.088 (1.043 – 1.134)	**<0.0001**	10157/106075	1.147 (1.109 – 1.187)	**<0.0001**
Q3	0.0527 (0.0119)	**<0.0001**	14252/106075	1.199 (1.156 – 1.243)	**<0.0001**	6593/106075	1.129 (1.074 – 1.186)	**<0.0001**	11515/106075	1.218 (1.170 – 1.268)	**<0.0001**
Q4	0.1430 (0.0124)	**<0.0001**	17622/106075	1.325 (1.276 – 1.375)	**<0.0001**	8719/106075	1.270 (1.208 – 1.336)	**<0.0001**	14317/106075	1.347 (1.293 – 1.404)	**<0.0001**
PhenoAge acceleration (Continuous)	0.1134 (0.0033)	**<0.0001**	54554/424299	1.149 (1.139 – 1.160)	**<0.0001**	26424/424299	1.138 (1.124 – 1.151)	**<0.0001**	43544/424299	1.156 (1.145 – 1.168)	**<0.0001**
**PhenoAge acceleration (Quartiles)**											
Q1	Ref		11113/106074	Ref		5092/106074	Ref		8792/106074	Ref	
Q2	0.0174 (0.0088)	**0.047**	11758/106075	1.030 (1.001 – 1.059)	**0.043**	5549/106075	1.040 (1.000 – 1.082)	0.05	9264/106075	1.025 (0.994 – 1.058)	0.12
Q3	0.0659 (0.0089)	**<0.0001**	13389/106075	1.111 (1.080 – 1.143)	**<0.0001**	6530/106075	1.130 (1.087 – 1.176)	**<0.0001**	10586/106075	1.106 (1.073 – 1.141)	**<0.0001**
Q4	0.2267 (0.0093)	**<0.0001**	18294/106075	1.340 (1.304 – 1.378)	**<0.0001**	9253/106075	1.334 (1.283 – 1.386)	**<0.0001**	14902/106075	1.361 (1.320 – 1.403)	**<0.0001**

^a^Model adjusted for age, sex, ethnic, BMI, smoking status, healthy alcohol intake, healthy physical activity, hypertension, diabetes, coronary heart disease, and Townsend deprivation index. The examination center was controlled for a random effect. Two-sided statistical tests were conducted and no adjustments were made for multiple comparisons. Bolded *p*-values are statistically significant (<0.05).

### Biological aging and incident depression/anxiety over follow-up

**Fig. 2:**
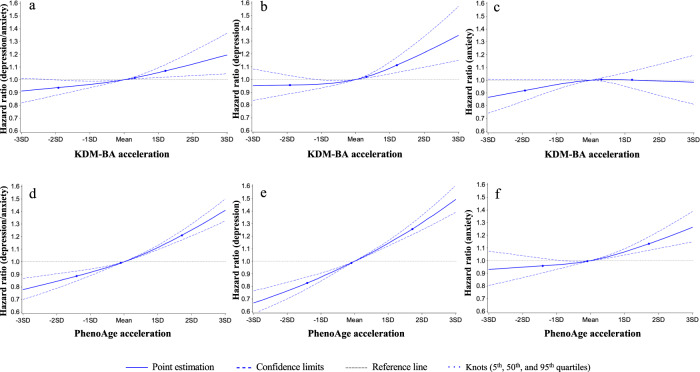
Graphs of the best fitting models for relationships of KDM-BA acceleration and PhenoAge acceleration with incident depression/anxiety disorders at follow-up. Panels: KDM-BA acceleration (**a**–**c**) and PhenoAge acceleration (**d**–**f**). Solid line: Point estimation; Black dash line: Confidence limits; Green dash line: Reference line; Dots: Knots (5th, 50th, and 95th percentiles). Restricted cubic spline regression model adjusted for age, sex, BMI category, race, smoking status (current/former/never), healthy alcohol intake status, healthy physical activity status, Townsend deprivation index, and prevalent hypertension, CHD, and diabetes. Source data are provided as a Source Data file.

To test if older biological age predisposed to incident depression/anxiety, we analyzed data for the subset of 369,745 participants who were free of depression and anxiety at baseline. Older biological age was associated with increased risk of incident depression/anxiety over a median 8.7 years of follow-up. Effect-sizes were largest for depression and smallest for anxiety; for KDM-BA, each SD change in its acceleration was associated with a 4–7% increase in risk of incident depression, anxiety, or either disorder; for PhenoAge acceleration, risk increments per SD were 6–14% (Table 3 and S2). In dose-response analysis, we found that risk for all outcomes increased monotonically with higher PhenoAge acceleration. Patterns were more mixed for KDM-BA acceleration (Table 3, Fig. 2). Finally, we tested if older biological age was associated with the incidence of comorbid depression and anxiety. It was; however, the association was statistically different from zero only for the PhenoAge acceleration [per SD increment in risk=9.3% (95% CI: 5.4–13.2%); Table 4].

**Table 3:** Associations of biological ages at baseline with incident depression/anxiety disorders at follow-up (fully adjusted model)^a^

Biological age	Incident depression/anxiety disorders			Incident depression			Incident anxiety		
	N_case_/N_total_	Hazard ratio (95% CI)	*p*-value	N_case_/N_total_	Hazard ratio (95% CI)	*p*-value	N_case_/N_total_	Hazard ratio (95% CI)	*p*-value
KDM-BA acceleration (Continuous)	16523/369745	1.059 (1.033 – 1.085)	**<0.0001**	11042/369745	1.070 (1.039 – 1.103)	**<0.0001**	8472/369745	1.038 (1.002 – 1.075)	**0.037**
**KDM-BA acceleration (Quartiles)**									
Q1	2858/92436	Ref		1924/92436	Ref		1442/92436	Ref	
Q2	3952/92436	1.103 (1.035 – 1.175)	**0.0024**	2653/92436	1.077 (0.997 – 1.163)	0.06	2012/92436	1.056 (0.961 – 1.159)	0.26
Q3	4696/92437	1.105 (1.048 – 1.166)	**0.0002**	3005/92437	1.113 (1.043 – 1.187)	**0.0012**	2538/92437	1.107 (1.013 – 1.211)	**0.025**
Q4	5017/92436	1.125 (1.053 – 1.201)	**0.0005**	3460/92436	1.161 (1.073 – 1.257)	**0.0002**	2480/92436	1.083 (1.004 – 1.169)	**0.039**
PhenoAge acceleration (Continuous)	16523/369745	1.113 (1.096 – 1.130)	**<0.0001**	11042/369745	1.145 (1.125 – 1.167)	**<0.0001**	8472/369745	1.063 (1.040 – 1.087)	**<0.0001**
**PhenoAge acceleration (Quartiles)**									
Q1	3726/92436	Ref		2289/92436	Ref		2095/92436	Ref	
Q2	3753/92436	1.032 (0.985 – 1.080)	0.18	2445/92436	1.075 (1.015 – 1.139)	**0.014**	2021/92436	1.012 (0.951 – 1.076)	0.71
Q3	4152/92437	1.132 (1.082 – 1.185)	**<0.0001**	2823/92437	1.209 (1.143 – 1.280)	**<0.0001**	2085/92437	1.058 (0.994 – 1.127)	0.07
Q4	4892/92436	1.286 (1.228 – 1.346)	**<0.0001**	3485/92436	1.405 (1.328 – 1.487)	**<0.0001**	2271/92436	1.151 (1.079 – 1.226)	**<0.0001**

^a^Analyses were conducted in 369,745 participants free of depression/anxiety at baseline; Model adjusted for age, sex, ethnic, BMI, smoking status, healthy alcohol intake, healthy physical activity, hypertension, diabetes, coronary heart disease, and Townsend deprivation index. The examination center was controlled for as a random effect. Two-sided statistical tests were conducted and no adjustments were made for multiple comparisons. Bolded *p*-values are statistically significant (<0.05).

**Table 4:** Associations of biological ages at baseline with co-incident depression and anxiety at follow-up^a^

Biological ages	Co-incident depression and anxiety		
	N_case_/N_total_	Hazard ratio (95% CI)	*p*-value
KDM-BA acceleration (Continuous)	2991/369745	1.038 (0.979 – 1.101)	0.21
**KDM-BA acceleration (Quartiles)**			
Q1	508/92436	Ref	
Q2	713/92436	1.019 (0.877 – 1.185)	0.80
Q3	847/92437	1.043 (0.892 – 1.220)	0.60
Q4	923/92436	1.064 (0.938 – 1.207)	0.34
PhenoAge acceleration (Continuous)	2991/369745	1.093 (1.054 – 1.132)	**<0.0001**
**PhenoAge acceleration (Quartiles)**			
Q1	658/92436	Ref	
Q2	713/92436	1.107 (0.995 – 1.231)	0.06
Q3	756/92437	1.159 (1.042 – 1.289)	**0.0066**
Q4	864/92436	1.282 (1.152 – 1.427)	**<0.0001**

^a^Analyses were conducted in 369,745 participants free of depression/anxiety at baseline; the Model was adjusted for age, sex, ethnic, BMI, smoking status, healthy alcohol intake, healthy physical activity, hypertension, diabetes, coronary heart disease, and Townsend deprivation index. The examination center was controlled for a random effect. Two-sided statistical tests were conducted and no adjustments were made for multiple comparisons. Bolded *p*-values are statistically significant (<0.05).

**Fig. 3:**
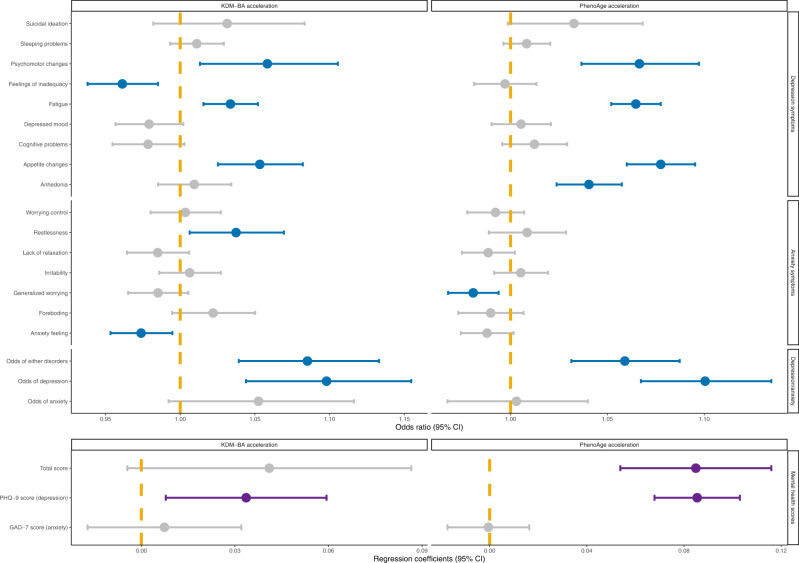
Prospective associations of baseline biological age accelerations with odds of depression/anxiety symptoms and mental health scores at follow-up survey for the 124,976 participants that were free of depression and anxiety at baseline with available online mental health survey data. Dots (centers of error bars): Point estimate; Error bar: 95% confidence limits; Dash line: Reference line; Upper part is the odds ratios of logistic regression, lower part is the coefficients of linear regression; Dots and error bars colored in blue (for odds ratios) or purple (for coefficients) are statistically significant (unadjusted *p*-values < 0.05), otherwise are colored in grey. The logistic regression model was used in analyses for the odds of depression/anxiety symptoms and the linear regression model was used for the mental health scores. Model adjusted for age, sex, BMI category, race, smoking status (current/former/never), healthy alcohol intake status, healthy physical activity status, Townsend deprivation index, and prevalent hypertension, CHD, and diabetes. The examination center was additionally controlled for a random effect. Two-sided statistical tests were conducted and no adjustments were made for multiple comparisons. Source data are provided as a Source Data file.

We analyzed the associations of the two AAs with symptoms of depression/anxiety in the subset of participants who were free of depression/anxiety at baseline and who participated in the follow-up survey (*N* = 124,976). Both AAs demonstrated positive associations with the odds of incident depression, anxiety, or either disorder. Participants with older KDM-BA showed a higher incidence of three depression symptoms: psychomotor changes, fatigue, and appetite changes (Fig. 3). The three symptoms were positively correlated with each other (correlation coefficients: 0.17–0.33, Table S3). Higher PhenoAge acceleration was associated with the risk of newly diagnosed depression and with the incidence of the three depression symptoms plus anhedonia. Baseline levels of biological age were not consistently associated with most symptoms of anxiety. They were positively associated with increased symptom scores of either disorder, depression, and anxiety, but associations with the anxiety score were not statistically significant.

We conducted four further sensitivity analyses. First, we tested if associations of biological aging measures with incident depression/anxiety varied between participants according to their chronological age and sex (*N* = 369,745). We observed no differences in associations between chronologically younger and older participants. However, there was some evidence of a sex difference; for PhenoAge acceleration, associations were somewhat stronger for men as compared with women (Table S4). Second, we repeated the analysis of incidence restricting the sample to participants with >2 years of follow-up. In this sensitivity analysis, both measures of biological aging showed robust associations with incident depression/anxiety (Table S5). Third, we considered whether older biological age might contribute to depression and anxiety through its effects on the incidence of other chronic diseases. People with incident depression/anxiety experienced higher incidence of chronic conditions over follow-up as compared to those who did not have incident depression/anxiety (for diabetes, 5.8% vs. 2.9%; for cardiovascular diseases, 11.6% vs. 5.9%; for cancers, 11.1% vs. 7.9%; all *p-values* < 0.0001; Table S6). To probe whether the process through which older biological age might contribute to risk of depression/anxiety could be mediated by the psychological burdens of chronic disease, we repeated our original incidence analysis including a covariate for whether the individual diagnosed with incident diabetes, cardiovascular disease, or cancer during the follow-up interval. The effect-sizes were attenuated but remained statistically different from zero at the alpha=0.05 threshold (Table S7). Last, we considered whether childhood adversity might explain associations of older biological age with depression/anxiety. UK Biobank collected reports of childhood adversity as part of the follow-up online survey. Therefore, we repeated the analysis of incident depression/anxiety in the subset of participants who were free of depression/anxiety at baseline and who participated in the follow-up survey (*N* = 124,976). In models including covariate adjustment for childhood adversity, effect-sizes for biological age measures were similar to effect-sizes from our original analyses of incident depression/anxiety (Table S8).

### Joint effects of biological age accelerations and genetic susceptibility

**Fig. 4:**
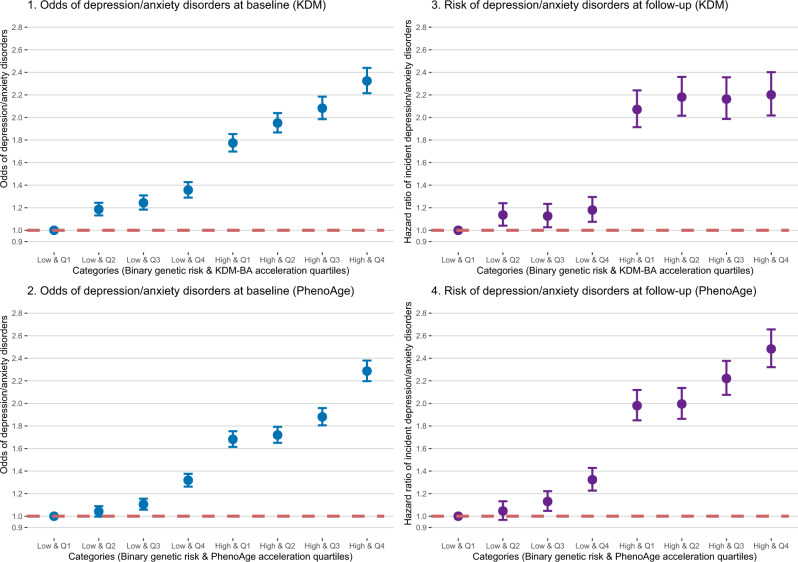
Joint associations of genetic risk and biological age accelerations with the odds of depression/anxiety disorders at baseline and incident depression/anxiety disorders at follow-up. Dots (centers of error bars): Point estimate; Error bar: 95% confidence limits; Dash line: Reference line; Logistic regression model was used in analyses for the odds of depression/anxiety at baseline and Cox regression model was used for the risk of incident depression/anxiety during the follow-up. Model adjusted for age, sex, BMI category, race, smoking status (current/former/never), healthy alcohol intake status, healthy physical activity status, Townsend deprivation index, and prevalent hypertension, CHD, and diabetes. The examination center was additionally controlled for as a random effect. Two-sided statistical tests were conducted and no adjustments were made for multiple comparisons. Source data are provided as a Source Data file.

Finally, we tested whether background genetic risk for depression/anxiety disorders combined in additive or synergistic ways with biological aging to influence risk for depression/anxiety. We measured genetic risk using a polygenic risk score (PRS) defined from two genome-wide-association studies. Participants with higher levels of genetic risk as measured by the PRS tended to have poorer mental health at baseline (Table S9) and were more likely to have incident disorder over follow-up (Table S10). However, participants’ genetic risk was not related to their biological aging (*p*-values>0.05; Table S10). We next evaluated risk synergy between genetic risk and biological age by testing interactions between the PRS and the biological age measures in models predicting prevalent and incident depression/anxiety (Table S11). Genetic risk and biological aging contributed independently and additively to risk for depression/anxiety; there was no robust evidence for risk synergy (*p*-values for interaction ranges 0.047–0.75). Joint effects of genetic risk and biological age are reported in Table S12 and graphed in Fig. 4. For instance, participants with the highest levels of both genetic risk and biological age were with nearly 2.5 times risk of incident depression/anxiety during follow-up (hazard ratio [HR] = 2.483, 95% CI: 2.321–2.656) as compared with those at the lowest levels of genetic risk and biological age.

## Discussion

In this study, we tested associations of blood-chemistry measures of biological aging with prevalent and incident depression and anxiety among a half-million midlife and older adults in the UK Biobank. The main findings were that adults with more advanced biological age were more likely to experience depression and anxiety at baseline and were at higher risk of depression/anxiety over eight years of follow-up, as compared with peers who were the same chronological age, but who were tested to be biologically younger. The risk associated with biological age was independent of and additive to genetic risk measured using a PRS. The risk was also independent of self-reported history of childhood adversity. This study contributes evidence from a large biobank cohort to support the hypothesis that biological aging might represent a risk factor for depression/anxiety in midlife and older adults. Findings suggest future directions for depression/anxiety risk assessment in older adults as well as the potential for therapies that target the biology of aging to contribute to the prevention of later-life depression/anxiety.

There is accumulating evidence for a link between mental health problems and biological aging^6,7,9,22,23^. However, most studies have focused on poor mental health as a risk factor for accelerated aging. The reverse process may also occur. For example, white matter hyperintensities, neuroimaging signatures of small cerebral infarcts, are associated with aging and with the risk of depression^24^, and recently have been linked to measurements of biological aging^25^. The same is true of low-grade systemic inflammation and mitochondrial dysfunction^26–30^. Consistent with this complementary hypothesis of biological aging as a risk factor for depression/anxiety, older adults in the Health, Aging, and Body Composition Study who were tested to have older biological age using the KDM measure tended to report more prevalent and incident symptoms of depression on the Center for Epidemiologic Studies Depression Scale (CES-D)^9^. The data reported here extend those observations to clinical diagnosis of depression in a very large sample with a broader age range.

Our findings help establish a prospective link connecting older biological age with incident depression/anxiety. But they do not address the mechanisms mediating this link, which could be formed at multiple stages in the progression of aging processes from accumulating molecular alterations to physical impairments and chronic disease. One hypothesis is that molecular changes at the roots of biological aging, including telomere attrition and mitochondrial dysfunction, have direct effects on psychological processes contributing to depression and anxiety^31,32^. A parallel hypothesis suggests that physiological changes downstream from the molecular roots of aging, including cerebrovascular alterations, could trigger depression or anxiety^26^. Or, yet further downstream of aging biology, poor physical health may itself impair psychological well-being and contribute to psychiatric disorder^33^. In our study, individuals with incident depression/anxiety also experienced a higher incidence of diabetes, cardiovascular diseases, and cancers as compared with those who were free of depression/anxiety at baseline and follow-up. Including covariate adjustment for comorbid chronic disease incidence attenuated biological age associations with depression/anxiety, suggesting that the development of physical health problems may partly mediate the link between older biological age and risk for incident depression/anxiety. Additionally, the risk associated with biological age was independent of childhood adversity, which suggesting that the relationship between biological aging and late-life depression/anxiety are not confounded by the common cause of childhood adversity.

The evidence we report for prospective associations between older biological age and incident depression/anxiety do not rule out a parallel casual pathway in the opposite direction. Instead, combined with prospective studies that link poor mental health with older biological age and faster pace of biological aging^31,32^, our findings suggest the possibility of reciprocal interactions between biological aging and psychiatric morbidity in which each reinforces the other. To address questions of mechanism and to illuminate patterns of reverse causation or reciprocal interaction between biological aging and psychiatric morbidity, data are needed that contain repeated assessments of biological aging and its sequelae at multiple levels (i.e., cellular, tissue/organ, and whole-patient) along with assessments of psychiatric symptoms. Prospective cohort studies are beginning to develop this data architecture, e.g., several cohort studies within the Gateway to Global Aging (https://g2aging.org/) are now incorporating molecular and physiological assessments to complement detailed social and behavioral assessments.

We acknowledge limitations. Foremost, our study is observational. While we can establish a prospective association between older biological age and depression/anxiety, causality remains uncertain. The data reported here do not rule out the possibility that older biological age is associated with risk for depression/anxiety because of unobserved shared causes. We did observe that older biological age was associated with incident depression/anxiety in adults free of both disorders at baseline. And we were able to rule out confounding by genetic risk for both disorders. However, it is possible that signs of more advanced biological aging simply emerge before the symptoms of depression/anxiety. Longitudinal, repeated-measure studies that can measure both biological aging and mental disorder at multiple time points can help to address this limitation by testing if more pronounced increases in biological age with the passage of time are reflected in increases in risk for depression/anxiety. In parallel, analysis of biological age in trials of anti-depressants and other therapies can clarify whether reductions in depression/anxiety can slow biological aging^2^. As a volunteer cohort, participants in the UK Biobank do not represent the UK population, tending to be healthier and wealthier^34,35^. Only about 1/3 of enrolled participants participated in the follow-up survey. The sample we analyzed may therefore have younger biological age and exhibit lower levels of depression/anxiety than the general population, and/or reflect a different distribution of causes of these outcomes. We may also lose older individuals who become ill to participate or who die during follow-up. However, we anticipate these selection biases will attenuate association estimates toward the null. Our results are therefore conservative. Depression and anxiety diagnoses were obtained from hospital records and questionnaires. Many individuals affected by either disorder do not seek treatment^36^, and those with other undiagnosed mental disorders may further lead to depression/anxiety. However, this ascertainment bias and hidden mental disorders are expected to bias our estimates of association toward the null; our effect-size estimates are therefore conservative. The same is true for our analysis of genetic confounding; the GWAS from which the depression polygenic index was derived included UK Biobank, which will bias upward associations between this measure and depression, and could also inflate covariation between the PRS and our measures of bological aging ^37^. Additionally, our participants were mostly (~95%) middle-aged or older white adults, which limits the generalization of the results to other age and ethnic groups. Finally, our analysis that found early-life adversity did not explain associations between older biological age and increased risk of depression/anxiety relied on retrospective reports of early-life diversity. Recall bias could limit the power of this analysis.

In conclusion, we found that adults with older biological ages showed an increased risk for prevalent and incident depression/anxiety disorders. Under the circumstances of population aging, increased attention to the prevention and treatment of depression/anxiety disorders in older adults is a public health priority^26^. Interventions to slow biological aging, beginning from midlife, may represent on the path to reducing the burden of disease.

## Methods

### Study design and population

UK Biobank research has received approval from the North West Multicenter Research Ethical Committee. Written informed consent were provided by all participants. UK Biobank is an ongoing prospective study with 502,536 participants recruited in 2006–2010 at the age of 37–73 years (baseline survey) with multiple follow-ups^38^. At baseline, participants were asked to provide information on their lifestyle and health, and their biological samples were collected. During 2016–2017, the mental health status of ~1/3 of participants was obtained via an online survey (i.e., follow-up survey). In this study (Figure S1), we included 424,299 participants with available data on mental health status, measures of traits for included in biological age algorithms, potential covariates, and genetic variants at baseline. After excluding 54,554 participants with depression/anxiety at baseline, we conducted another two analyses in the remaining 369,745 participants who were free of depression or anxiety at baseline. A total of 369,745 participants who were free of depression or anxiety at baseline were employed to assess the association between biological aging and the incidence of depression and/or anxiety. A subset of 124,976 of the 369,745 participants reported their mental health status at the follow-up survey were used to evaluate the prospective associations between baseline biological aging and the syndromes of depression or anxiety in between.

### Assessment of depression and anxiety symptoms

Assessments of depression and anxiety symptoms were conducted using both linked hospital admission records (UK Biobank Data-Fields: 41202 and 41204) and mental health questionnaires at baseline (for prevalent disorders) and follow-up (for incident disorders). For hospital records, participants were classified as with depression or anxiety symptoms if they had either an ICD-9/10 code of primary or secondary diagnosis for depression (ICD-9: 311; ICD-10: F32-F33) or anxiety (ICD-9: 300; ICD-10: F40-F41).

For mental health questionnaire screening, at baseline, depression, and anxiety symptoms were assessed by Patient Health Questionnaire (PHQ)-4 questionnaire^39^. Participants were required to rate, on a four-point Likert scale from 0 (not at all) to 3 (nearly every day), their response to four items: 1. “frequency of depressed mood” (Data-Field: 2050), 2. “frequency of unenthusiasm/disinterest” (Data-Field: 2060), 3. “frequency of tenseness/restlessness” (Data-Field: 2070), and 4. “frequency of tiredness/lethargy” (Data-Field: 2080). The total scores ranged from 0 to 12, and a score of ≥6 was considered positive for depression/anxiety disorders. A total score of ≥3 for items 1 and 2 was considered as positive for depression, and a total score of ≥3 for items 3 and 4 was considered as positive for anxiety. At the follow-up survey, mental health status was assessed using PHQ-9^40^ and Generalized Anxiety Disorder (GAD)-7^41^ questionnaires between 2016 and 2017 with the same four-point Likert scale from 0 (not at all) to 3 (nearly every day). PHQ-9 consists of 9 items: 1. “recent thoughts of suicide or self-harm” (Suicidal ideation; Data-Field: 20513), 2, “trouble falling or staying asleep, or sleeping too much” (Sleeping problems; Data-Field: 20517), 3. “recent changes in speed/amount of moving or speaking” (Psychomotor changes; Data-Field: 20518), 4. “recent feelings of inadequacy” (Feelings of inadequacy; Data-Field: 20507). 5. “recent feelings of tiredness or low energy” (Fatigue; Data-Field: 20519), 6. “recent feelings of depression” (Depressed mood; Data-Field: 20510), 7. “recent trouble concentrating on things” (Cognitive problems; Data-Field: 20508), 8. “recent poor appetite or overeating” (Appetite changes; Data-Field: 20511), and 9. “recent lack of interest or pleasure in doing things” (Anhedonia; Data-Field: 20514). GAD-7 consists of 7 items: 1. “recent inability to stop or control worrying” (Worrying control; Data-Field: 20509), 2. “recent restlessness” (Restlessness; Data-Field: 20516), 3. “recent trouble relaxing” (Lack of relaxation; Data-Field: 20515), 4. “recent easy annoyance or irritability” (Irritability; Data-Field: 20505), 5. “recent worrying too much about different things” (Generalized worrying; Data-Field: 20520), 6. “recent feelings of foreboding” (Foreboding; Data-Field: 20512), and 7. “recent feelings of nervousness or anxiety” (Anxiety feeling; Data-Field: 20506). Any item with a score of ≥1 was considered positive for this symptom. A PHQ-9 or GAD-7 total score of ≥10 was considered depression or anxiety symptoms positive. Other available assessments including the Composite International Diagnostic Interview Short Form (CIDI-SF) was not selected^42^.

Analysis 2 used both the assessments from the online follow-up mental health survey and the hospital records. Analysis 3 included a subset of participants with the data from the online follow-up mental health survey. For participants free of depression and anxiety at baseline, their follow-up time ended on the date of hospital records of incident depression/anxiety disorders, the date of follow-up survey, or the date of censoring (Dec 31st 2018), whichever occurred first.

### Assessment of biological ages and age accelerations

We measured biological age using the best-validated algorithms that could be implemented with data available in the UK Biobank, the KDM-BA and the PhenoAge^16–18^, using blood-chemistry-derived measures^43^. Briefly, KDM-BA was computed from forced expiratory volume in one second (FEV_1_), systolic blood pressure, and seven blood chemistry parameters (albumin, alkaline phosphatase, blood urea nitrogen, creatinine, C-reactive protein, glycated hemoglobin, and total cholesterol); PhenoAge was computed from nine blood chemistries including four overlapped with KDM-BA (albumin, alkaline phosphatase, creatinine, C-reactive protein, glucose, mean cell volume, red cell distribution width, white blood cell count, and lymphocyte proportion). Included biomarkers and corresponding UK Biobank data-fields are reported in Table 1 and S13. Any observations with missing values were excluded from this analysis (Fig. S1). Computation of biological age values was conducted using the R package ‘BioAge’ (https://github.com/dayoonkwon/BioAge)^44^.

An individual’s KDM-BA prediction corresponds to the chronological age at which her/his physiology would be approximately normal. The KDM-BA is derived from a series of regressions of individual biomarkers on chronological age in a reference population. The equation takes information from *n* number of regression lines of chronological age regressed on *n* biomarkers^44^. The formula is:

$${{KDM}-{BA}}_{{EC}}=\frac{{\sum }_{{{\mbox{i}}}=1}^{n}\left({x}_{{{\mbox{i}}}}-{q}_{{{\mbox{i}}}}\right)\frac{{k}_{{{\mbox{i}}}}}{{s}_{{{\mbox{i}}}}^{2}}+\frac{{CA}}{{s}_{{BA}}^{2}}}{{\sum }_{{{\mbox{i}}}=1}^{n}{\left(\frac{{k}_{{{\mbox{i}}}}}{{s}_{{{\mbox{i}}}}}\right)}^{2}+\frac{1}{{s}_{{BA}}^{2}}}$$

where x is the value of biomarker i measured for an individual. For each biomarker i, the parameters k, q, and s are estimated from a regression of chronological age on the biomarker in the reference sample. k, q, and s are the regression intercept, slope, and root mean squared error, respectively. sBA is a scaling factor equal to the square root of the variance in chronological age explained by the biomarker set in the reference sample. CA is chronological age. In the BioAge package, the reference sample is NHANES III nonpregnant participants aged 30–75 years. Algorithm parameters are estimated separately for men and women. In our study, we used nine biomarkers including forced expiratory volume in one second (FEV_1_), systolic blood pressure, albumin, alkaline phosphatase, blood urea nitrogen, creatinine, C-reactive protein, glycated hemoglobin, and total cholesterol.

The PhenoAge algorithm is derived from multivariate analysis of mortality hazards. The original PhenoAge algorithm was constructed from elastic-net Gompertz regression of mortality on 42 biomarkers in the NHANES III. This analysis selected nine biomarkers: albumin, alkaline phosphatase, creatinine, C-reactive protein, glucose, mean cell volume, red cell distribution width, white blood cell count, and lymphocyte proportion, and chronological age. The formula is:

$${{\mbox{PhenoAge}}}=141.50225+\frac{{{{{{\rm{ln}}}}}}\left[-0.00553\times {{{{{\rm{ln}}}}}}(1-{{{{{\rm{mortality}}}}}}\,{{{{{\rm{risk}}}}}})\right]}{0.090165}$$

where

$${{\mbox{mortality}}}\,{{\mbox{risk}}}=1-{e}^{-{e}^{{{xb}}_{\left[\exp ({120}_{x\gamma })-1\right]/\gamma }}}$$

$$\gamma=0{{\mbox{.}}}0076927$$

$$xb=	-19.907-0.0336+{{{{{\rm{albumin}}}}}}+0.0095 \times {{{{{\rm{creatinine}}}}}}+0.1953\\ 	 \times {{{{{\rm{glucose}}}}}}+0.0954\times \,{{{{\mathrm{ln}}}}}(C-{{{{{\rm{reactive}}}}}}\,{{{{{\rm{protein}}}}}})-0.012\\ 	 \times {{{{{\rm{lymphocyte}}}}}}\,{{{{{\rm{percentage}}}}}}+0.0268\times {{{{{\rm{mean}}}}}}\,{{{{{\rm{corpuscular}}}}}}\,{{{{{\rm{volume}}}}}}\\ 	+0.3306 \times {{{{{\rm{red}}}}}}\,{{{{{\rm{cell}}}}}}\,{{{{{\rm{distribution}}}}}}\,{{{{{\rm{width}}}}}}+0.00188\times {{{{{\rm{alkaline}}}}}}\,{{{{{\rm{phosphatase}}}}}}\\ 	+0.0554\times {{{{{\rm{white}}}}}}\,{{{{{\rm{blood}}}}}}\,{{{{{\rm{cell}}}}}}\,{{{{{\rm{count}}}}}}+0.0804\times {{{{{\rm{chronological}}}}}}\,{{{{{\rm{age}}}}}}$$

To quantify differences between participants in biological aging, we regressed their computed biological age values on their chronological ages at the time of biomarker measurement and computed the residual values. We hereafter refer to these residuals as “age acceleration (AA)” values to gauge biological aging. In order to make effect-sizes for the two measures of biological aging comparable, we standardized the AAs to have a mean value of 0 and standard deviation (SD) of 1 for analysis of a continuous dimension and to quartiles for dose-response analysis.

### Measurements of covariates

We included age, sex, body mass index (BMI), race (Whiter, Black, Asian, and other), smoking status (current/former/never), healthy alcohol intake status, healthy physical activity status, Townsend deprivation index (continuous), prevalent hypertension, coronary heart disease (CHD), and diabetes as potential covariates in this study. Height and weight were measured by trained nurses during the baseline assessment center visit, and BMI was calculated by dividing weight in kilograms by the square of height in meters and then was classified into: underweight or normal weight (<25), overweight (25 to <30), and obese (≥30). Healthy alcohol intake status was defined as: male: <28 g/day; female: <14 g/day. Healthy physical activity status was defined as: ≥150 min/week moderate or ≥75 min/week vigorous or 150 min/week mixed (moderate + vigorous) activity. Physical activity was assessed using the Metabolic Equivalent Task minutes based on adopted items from the short International Physical Activity Questionnaire^45^. Townsend deprivation index is constructed based on four key variables in UK Biobank: unemployment, overcrowded household, non–car ownership, and non–home ownership, a higher index indicates a higher level of deprivation. History of hypertension, CHD, and diabetes was based on self-reported information and medical records at baseline and during the follow-up. Additional participants with ≥140 mmHg SBP and/or ≥90 mmHg SBP at the enrollment were also included as individuals with hypertension at baseline. Observations with any missing values of the covariates were excluded from this study. We also collected their incident diabetes, cardiovascular diseases, and cancer^46^ to examine whether incident depression/anxiety cases had higher probabilities of having major chronic physical health disorders than the healthy.

### Assessment of childhood adversity

Because childhood adversity may affect biological aging and relevant health outcomes^47^. We tested whether childhood adversity could affect the associations between biological aging and incident depression/anxiety disorders among 124,976 baseline depression/anxiety-free participants with available data. Childhood adversity, sourced from the follow-up survey, was assessed with five questions representing physical neglect, emotional neglect, sexual abuse, physical abuse, and emotional abuse, using the Childhood Trauma Screener, which is a shortened version of the Childhood Trauma Questionnaire and is a cost-efficient, validated, and relatively reliable screening tool in large epidemiological studies^48,49^: (1) felt hated by a family member (emotional abuse, Data-Field: 20487); (2) physically abused by family as a child (physical abuse, Data-Field: 20488); (3) felt loved as a child (emotional neglect, Data-Field: 20489); (4) sexually molested as a child (sexual abuse, Data-Field: 20490); and (5) someone to take to the doctor when needed as a child (physical neglect, Data-Field: 20491).

For each question, potential responses included never true, rarely true, sometimes true, often true, and very often true. Physical neglect was dichotomized as 1 if participants answered never true, rarely true, sometimes true, or often true; emotional neglect was dichotomized as 1 if participants answered never true, rarely true, or sometimes true; sexual abuse, physical abuse, and emotional abuse were dichotomized as 1 if participants answered rarely true, sometimes true, often true, and very often true. The summary score of 5 items ranged from 0 to 5, with a higher score denoting more childhood adversities.

### Polygenic risk scores for depression and anxiety

We measured background genetic risk for depression/anxiety disorders using PRS for depression, anxiety, and a combined depression/anxiety phenotype. PRS were computed based on results from the largest published genome-wide association studies (GWASs) of depression and anxiety^37,50^. We also conducted a meta-analysis of the two GWASs using METAL software to produce a GWAS of a combined depression/anxiety phenotype^51^. Detailed information about the genotyping, data imputation, and quality control in the UK Biobank has been reported^38,52^. Briefly, we excluded SNPs with low minor allele frequency (MAF) (MAF < 1%) or imputation information scores (INFO) (INFO < 0.8). Mismatched, duplicated, and ambiguous SNPs were also excluded. We computed participants PRS by combining the UK Biobank SNP database with summary statistics from the GWAS using the PRSice-2 software V2.3.5^53^. The PRS was adjusted by sex and ten genetic principal components. The best-fitting parameters for the PRS for depression, anxiety, and both disorders were demonstrated in Table S14. The resulting PRSs explained of 7.7%~9.9% of variance in the odds of depression, and anxiety, either disorder, and the PHQ-4 score at baseline (Table S9).

### Statistical analysis

We first tested the cross-sectional associations of two AAs with PHQ-4 scores using mixed-effect linear regression models and with the odds of depression/anxiety disorders, depression, and anxiety at baseline using logistic regression (Analysis 1). We fit a series of models adjusting for increasing numbers of covariates: Model 1 adjusted for age and sex; Model 2 additionally adjusted for BMI category, race, smoking status, healthy alcohol intake, and healthy physical activity; Model 3 further adjusted for Townsend deprivation index, hypertension, CHD, and diabetes. Townsend deprivation index ranged from −6.3 to 10.2 is constructed based on four key variables in UK Biobank: unemployment, overcrowded household, non–car ownership, and non–home ownership; a higher index indicates a higher level of deprivation. The examination center was additionally controlled for in all models as a random effect to account for the potential residual bias from health examinations. Dose-response curves for associations with PHQ-4 scores were assessed by restricted cubic spline regression models controlling for all potential covariates^54^, with the 5th, 50th, and 95th percentiles of each AA selected as knots.

Furthermore, for the total participants free of depression and anxiety at baseline (Analysis 2), we used Cox proportional hazards model to test time-to-event associations of baseline AAs with the incident depression/anxiety over follow-up. Dose-response relationships related to the risk of incident depression/anxiety disorders, depression, and anxiety were assessed by restricted cubic spline regression. For Analysis 3, among participants free of depression and anxiety at baseline, we examined the prospective associations of the baseline AAs with the odds of newly identified depression/anxiety disorders and their symptoms (logistic regression), and the scores of PHQ-9 and GAD-7 (linear regression) in the subset of 124,976 participants with follow-up survey data. Models were adjusted for all covariates described.

Four sensitivity analyses were conducted. First, we tested whether age and sex could interact with both AAs in the prediction of depression/anxiety and the associations between AAs and the risk of comorbid depression and anxiety. Furthermore, we conducted another sensitivity analysis of Analysis 2 by excluding participants with ≤2 years of follow-up to avoid reversal causation. Third, we considered whether older biological age might contribute to depression and anxiety through its effects on the incidence of other chronic diseases. We additionally controlled for whether individuals were diagnosed with incident diabetes, cardiovascular diseases, or cancer during the follow-up in our primary model. Last, since adverse childhood experiences and childhood maltreatment are associated with older biological age in midlife adults and are risk factors for depression/anxiety across the life-course^55–57^, we examined whether childhood adversity could affect the associations between biological aging and incident depression/anxiety disorders among 124,976 baseline depression/anxiety-free participants with available childhood adversity data retrieved from follow-up survey.

Finally, to evaluate whether the genetic predisposition to depression/anxiety disorders may modify the association of biological aging with depression/anxiety disorders, we fitted models including terms of PRS and the interactions between PRS and AAs. In the case that the interaction effect did not meet the criteria for statistical significance (p-values<0.05), we generated a series of the categorical variables based on the quartiles of each AA and dichotomized PRS (by median) to assess the joint association of both factors with depression/anxiety.

SAS version 9.4 TS1M7 (SAS Institute Inc., Cary, NC, USA) was used to conduct data cleaning and analyses. A two-sided *p*-value of <0.05 was considered statistically significant.

### Reporting summary

Further information on research design is available in the Nature Portfolio Reporting Summary linked to this article.

### Supplementary information

Supplementary Information

Reporting Summary

### Source data

Source data

## Data Availability

Data are available in a public, open access repository. This research has been conducted using the UK Biobank Resource under Application Number 44430. The UK Biobank data are available on application to the UK Biobank (www.ukbiobank.ac.uk/) with access fees. Source data are provided with this paper.

## Code availability

The R code for biological age estimation in UK Biobank is published on https://zenodo.org/record/7777437. Other codes of SAS version 9.4 TS1M7 and R version 4.0 for statistical analysis of this study are available upon request from Dr. Xu Gao.
