# Author Correction: Accelerated biological aging and risk of depression and anxiety: evidence from 424,299 UK Biobank participants

**DOI:** 10.1038/s41467-023-41786-6

**Published:** 2023-09-25

**Authors:** Xu Gao, Tong Geng, Meijie Jiang, Ninghao Huang, Yinan Zheng, Daniel W. Belsky, Tao Huang

**Affiliations:** 1 Department of Occupational and Environmental Health Sciences, School of Public Health, Peking University, Beijing, China; 2 Peking University Sixth Hospital, Peking University Institute of Mental Health, NHC Key Laboratory of Mental Health (Peking University), National Clinical Research Center for Mental Disorders (Peking University Sixth Hospital), Beijing, China; 3 Department of Epidemiology and Biostatistics, School of Public Health, Peking University, Beijing, China; 4 Department of Preventive Medicine, Feinberg School of Medicine, Northwestern University, Chicago, IL USA; 5 Department of Epidemiology & Butler Columbia Aging Center, Columbia University, New York, NY USA

**Keywords:** Epidemiology, Depression, Predictive markers, Anxiety

Correction to: *Nature Communications* 10.1038/s41467-023-38013-7, published online 20 April 2023

The original version of this Article contained an error in the ‘Measurements of covariates’ part of the Methods section, which omitted the following sentence detailing inclusion criteria: ‘Additional participants with ≥140 mmHg SBP and/or ≥90 mmHg SBP at the enrolment were also included as individuals with hypertension at baseline’. This has been corrected in both the PDF and HTML versions of the Article.

