# Supplementary material for: Accelerated biological aging and risk of depression and anxiety: evidence from 424,299 UK Biobank participants
Source: Nat Commun. 2023 Apr 20;14:2277. doi: 10.1038/s41467-023-38013-7 (PMC10119095; doi:10.1038/s41467-023-38013-7)
Supplement: Supplementary file 1 — Supplementary Information [file 41467_2023_38013_MOESM1_ESM.pdf]

Supplementary Files

Table S1 Associations of biological ages with the PHQ-4 Score and odds of depression/anxiety disorders at baseline (Models 1 & 2)

Table S2 Associations of biological age accelerations at baseline with incident depression/anxiety disorders at follow-up (Models 1 & 2)

Table S3 Correlation matrix between psychomotor changes, fatigue, and appetite changes in 124,976 participants with online survey data

Table S4 Interactions between biological age accelerations and age/sex in predicting incident depression/anxiety disorders at follow-up

Table S5 Associations of biological ages at baseline with incident depression/anxiety disorders at follow-up among individuals with >2 years of follow-up

Table S6 Incident diabetes, cardiovascular diseases, and cancers by incident depression/anxiety status among 369,745 participants free of depression/anxiety at baseline

Table S7 Associations of biological age accelerations at baseline with incident depression/anxiety disorders at follow-up by additionally controlling for whether individuals diagnosed with incident diabetes, cardiovascular diseases, or cancer during the follow-up

Table S8 Associations of biological age accelerations at baseline with incident depression/anxiety disorders at follow-up among individuals with available follow-up survey data by additionally controlling for childhood adversity

Table S9 Associations of the odds of depression/anxiety disorders and PHQ-4 score with polygenetic risk scores at baseline

Table S10 Associations of polygenetic risk score with odds of depression/anxiety disorders at baseline, incident depression/anxiety disorders at follow-up, and the accelerations of two biological ages

Table S11 Mutual associations of biological age accelerations and polygenetic risk score with odds of depression/anxiety disorders at baseline and incident depression/anxiety disorders at follow-up

Table S12 Joint associations of polygenetic risk score and biological age accelerations with the depression/anxiety disorders at baseline and risks of incident depression/anxiety disorders during follow-up

Table S13 Full names and data field IDs of variables for the construction of biological ages

Table S14 Best-fitting parameters of the polygenetic risk scores for depression, anxiety, and both disorders

Figure S1 Flowchart for the selection of study participants and three subgroups for analysis

Figure S2 Correlation matrix of chronological age, biological ages, and age accelerations (Pearson correlation)

Table S1 Associations of biological ages with the PHQ-4 Score and odds of depression/anxiety disorders at baseline (Models 1 & 2) [a]

I. Model 1

Biological age	PHQ-4 Score		Depression/anxiety disorders			Depression			Anxiety		
	**Coefficients (SE)**	***p*-value**	**N_{case}/N_{total}**	**Odds ratio (95% CI)**	***p*-value**	**N_{case}/N_{total}**	**Odds ratio (95% CI)**	***p*-value**	**N_{case}/N_{total}**	**Odds ratio (95% CI)**	***p*-value**
KDM-BA acceleration (Continuous)	0.1866 (0.0042)	**<0.0001**	54554 / 424299	1.334 (1.318 – 1.351)	**<0.0001**	26424 / 424299	1.344 (1.322 – 1.366)	**<0.0001**	43544 / 424299	1.347 (1.329 – 1.366)	**<0.0001**
KDM-BA acceleration (Quartiles)											
Q1	Ref		9839 / 106074	Ref		4921 / 106074	Ref		7555 / 106074	Ref	
Q2	0.2064 (0.0097)	**<0.0001**	12841 / 106075	1.384 (1.343 – 1.426)	**<0.0001**	6191 / 106075	1.370 (1.315 – 1.427)	**<0.0001**	10157 / 106075	1.398 (1.352 – 1.446)	**<0.0001**
Q3	0.2862 (0.0118)	**<0.0001**	14252 / 106075	1.596 (1.541 – 1.653)	**<0.0001**	6593 / 106075	1.577 (1.503 – 1.654)	**<0.0001**	11515 / 106075	1.621 (1.559 – 1.685)	**<0.0001**
Q4	0.4924 (0.0117)	**<0.0001**	17622 / 106075	2.051 (1.983 – 2.121)	**<0.0001**	8719 / 106075	2.124 (2.030 – 2.223)	**<0.0001**	14317 / 106075	2.081 (2.005 – 2.160)	**<0.0001**
PhenoAge acceleration (Continuous)	0.2376 (0.0032)	**<0.0001**	54554 / 424299	1.322 (1.311 – 1.333)	**<0.0001**	26424 / 424299	1.323 (1.309 – 1.337)	**<0.0001**	43544 / 424299	1.331 (1.319 – 1.343)	**<0.0001**
PhenoAge acceleration (Quartiles)											
Q1	Ref		11113 / 106074	Ref		5092 / 106074	Ref		8792 / 106074	Ref	
Q2	0.0827 (0.0089)	**<0.0001**	11758 / 106075	1.118 (1.088 – 1.150)	**<0.0001**	5549 / 106075	1.137 (1.093 – 1.182)	**<0.0001**	9264 / 106075	1.116 (1.083 – 1.151)	**<0.0001**
Q3	0.2067 (0.0089)	**<0.0001**	13389 / 106075	1.324 (1.289 – 1.360)	**<0.0001**	6530 / 106075	1.373 (1.322 – 1.426)	**<0.0001**	10586 / 106075	1.323 (1.284 – 1.364)	**<0.0001**
Q4	0.5470 (0.0090)	**<0.0001**	18294 / 106075	1.959 (1.910 – 2.010)	**<0.0001**	9253 / 106075	2.043 (1.971 – 2.117)	**<0.0001**	14902 / 106075	2.004 (1.948 – 2.061)	**<0.0001**

II. Model 2

Biological age	PHQ-4 Score		Depression/anxiety disorders			Depression			Anxiety		
	**Coefficients (SE)**	***p*-value**	**N_{case}/N_{total}**	**Odds ratio (95% CI)**	***p*-value**	**N_{case}/N_{total}**	**Odds ratio (95% CI)**	***p*-value**	**N_{case}/N_{total}**	**Odds ratio (95% CI)**	***p*-value**
KDM-BA acceleration (Continuous)	0.0603 (0.0043)	**<0.0001**	54554 / 424299	1.145 (1.130 – 1.160)	**<0.0001**	26424 / 424299	1.131 (1.111 – 1.151)	**<0.0001**	43544 / 424299	1.157 (1.140 – 1.174)	**<0.0001**
KDM-BA acceleration (Quartiles)											
Q1	Ref		9839 / 106074	Ref		4921 / 106074	Ref		7555 / 106074	Ref	
Q2	0.0585 (0.0097)	**<0.0001**	12841 / 106075	1.169 (1.134 – 1.205)	**<0.0001**	6191 / 106075	1.130 (1.084 – 1.178)	**<0.0001**	10157 / 106075	1.182 (1.143 – 1.223)	**<0.0001**
Q3	0.0678 (0.0119)	**<0.0001**	14252 / 106075	1.239 (1.195 – 1.284)	**<0.0001**	6593 / 106075	1.185 (1.128 – 1.244)	**<0.0001**	11515 / 106075	1.259 (1.210 – 1.310)	**<0.0001**
Q4	0.1568 (0.0120)	**<0.0001**	17622 / 106075	1.377 (1.328 – 1.427)	**<0.0001**	8719 / 106075	1.354 (1.290 – 1.422)	**<0.0001**	14317 / 106075	1.398 (1.344 – 1.454)	**<0.0001**
PhenoAge acceleration (Continuous)	0.1420 (0.0033)	**<0.0001**	54554 / 424299	1.193 (1.183 – 1.204)	**<0.0001**	26424 / 424299	1.187 (1.174 – 1.202)	**<0.0001**	43544 / 424299	1.202 (1.190 – 1.214)	**<0.0001**
PhenoAge acceleration (Quartiles)											
Q1	Ref		11113 / 106074	Ref		5092 / 106074	Ref		8792 / 106074	Ref	
Q2	0.0194 (0.0088)	**0.028**	11758 / 106075	1.033 (1.005 – 1.063)	**0.023**	5549 / 106075	1.046 (1.005 – 1.088)	**0.028**	9264 / 106075	1.029 (0.997 – 1.061)	0.08
Q3	0.0745 (0.0090)	**<0.0001**	13389 / 106075	1.126 (1.095 – 1.158)	**<0.0001**	6530 / 106075	1.152 (1.108 – 1.198)	**<0.0001**	10586 / 106075	1.121 (1.087 – 1.156)	**<0.0001**
Q4	0.2854 (0.0093)	**<0.0001**	18294 / 106075	1.446 (1.407 – 1.487)	**<0.0001**	9253 / 106075	1.458 (1.404 – 1.515)	**<0.0001**	14902 / 106075	1.470 (1.426 – 1.515)	**<0.0001**

a: Model 1 adjusted for age and sex; Model 2 additionally adjusted for ethnic, BMI, smoking status, healthy alcohol intake, healthy physical activity, hypertension, diabetes, coronary heart disease, and Townsend deprivation index. The examination center was controlled for as a random effect in both models. Two-sided statistical tests were conducted and no adjustments were made for multiple comparisons. Bolded p-values are statistically significant (<0.05).

Table S2 Associations of biological age accelerations at baseline with incident depression/anxiety disorders at follow-up (Models 1 & 2) [a]

I. Model 1

Biological age	Incident depression/anxiety disorders			Incident depression			Incident anxiety		
	N_{case}/N_{total}	Hazard ratio (95% CI)	*p*-value	N_{case}/N_{total}	Hazard ratio (95% CI)	*p*-value	N_{case}/N_{total}	Hazard ratio (95% CI)	*p*-value
KDM-BA acceleration (Continuous)	16523 / 369745	1.152 (1.126 – 1.178)	**<0.0001**	11042 / 369745	1.193 (1.161 – 1.226)	**<0.0001**	8472 / 369745	1.097 (1.063 – 1.133)	**<0.0001**
KDM-BA acceleration (Quartiles)									
Q1	2858 / 92436	Ref		1924 / 92436	Ref		1442 / 92436	Ref	
Q2	3952 / 92436	1.209 (1.147 – 1.275)	**<0.0001**	2653 / 92436	1.249 (1.172 – 1.332)	**<0.0001**	2012 / 92436	1.149 (1.066 – 1.239)	**0.0003**
Q3	4696 / 92437	1.263 (1.188 – 1.343)	**<0.0001**	3005 / 92437	1.282 (1.190 – 1.382)	**<0.0001**	2538 / 92437	1.213 (1.112 – 1.323)	**<0.0001**
Q4	5017 / 92436	1.395 (1.313 – 1.482)	**<0.0001**	3460 / 92436	1.527 (1.419 – 1.643)	**<0.0001**	2480 / 92436	1.226 (1.124 – 1.337)	**<0.0001**
PhenoAge acceleration (Continuous)	16523 / 369745	1.188 (1.171 – 1.204)	**<0.0001**	11042 / 369745	1.243 (1.223 – 1.263)	**<0.0001**	8472 / 369745	1.107 (1.085 – 1.130)	**<0.0001**
PhenoAge acceleration (Quartiles)									
Q1	3726 / 92436	Ref		2289 / 92436	Ref		2095 / 92436	Ref	
Q2	3753 / 92436	1.071 (1.023 – 1.120)	**0.0033**	2445 / 92436	1.138 (1.075 – 1.205)	**<0.0001**	2021 / 92436	1.028 (0.976 – 1.093)	0.37
Q3	4152 / 92437	1.237 (1.183 – 1.293)	**<0.0001**	2823 / 92437	1.370 (1.296 – 1.448)	**<0.0001**	2085 / 92437	1.110 (1.044 – 1.180)	**0.0008**
Q4	4892 / 92436	1.550 (1.484 – 1.618)	**<0.0001**	3485 / 92436	1.803 (1.709 – 1.901)	**<0.0001**	2271 / 92436	1.285 (1.210 – 1.364)	**<0.0001**

II. Model 2

Biological age	Incident depression/anxiety disorders			Incident depression			Incident anxiety		
	N_{case}/N_{total}	**Hazard ratio (95% CI)**	***p*-value**	N_{case}/N_{total}	**Hazard ratio (95% CI)**	***p*-value**	N_{case}/N_{total}	**Hazard ratio (95% CI)**	***p*-value**
KDM-BA acceleration (Continuous)	16523 / 369745	1.066 (1.042 – 1.092)	**<0.0001**	11042 / 369745	1.073 (1.043 – 1.104)	**<0.0001**	8472 / 369745	1.053 (1.018 – 1.089)	**0.0026**
KDM-BA acceleration (Quartiles)									
Q1	2858 / 92436	Ref		1924 / 92436	Ref		1442 / 92436	Ref	
Q2	3952 / 92436	1.107 (1.040 – 1.178)	**0.0014**	2653 / 92436	1.073 (0.994 – 1.157)	0.07	2012 / 92436	1.089 (1.010 – 1.175)	**0.027**
Q3	4696 / 92437	1.107 (1.050 – 1.168)	**0.0002**	3005 / 92437	1.112 (1.043 – 1.185)	**0.0012**	2538 / 92437	1.124 (1.029 – 1.228)	**0.0094**
Q4	5017 / 92436	1.145 (1.075 – 1.220)	**<0.0001**	3460 / 92436	1.163 (1.077 – 1.255)	**0.0001**	2480 / 92436	1.099 (1.004 – 1.203)	**0.040**
PhenoAge acceleration (Continuous)	16523 / 369745	1.130 (1.113 – 1.147)	**<0.0001**	11042 / 369745	1.166 (1.145 – 1.187)	**<0.0001**	8472 / 369745	1.074 (1.051 – 1.098)	**<0.0001**
PhenoAge acceleration (Quartiles)									
Q1	3726 / 92436	Ref		2289 / 92436	Ref		2095 / 92436	Ref	
Q2	3753 / 92436	1.026 (0.980 – 1.075)	0.26	2445 / 92436	1.070 (1.010 – 1.133)	**0.022**	2021 / 92436	1.008 (0.947 – 1.072)	0.81
Q3	4152 / 92437	1.134 (1.084 – 1.187)	**<0.0001**	2823 / 92437	1.212 (1.145 – 1.283)	**<0.0001**	2085 / 92437	1.061 (0.997 – 1.129)	0.06
Q4	4892 / 92436	1.327 (1.268 – 1.388)	**<0.0001**	3485 / 92436	1.457 (1.378 – 1.542)	**<0.0001**	2271 / 92436	1.178 (1.105 – 1.255)	**<0.0001**

a: Analyses were conducted in 369,745 participants free of depression/anxiety at baseline; Model 1 adjusted for age and sex. Model 2 additionally adjusted for ethnic, BMI, smoking status, healthy alcohol intake, healthy physical activity, hypertension, diabetes, coronary heart disease, and Townsend deprivation index. The examination center was controlled for as a random effect in both models. Two-sided statistical tests were conducted and no adjustments were made for multiple comparisons. Bolded p-values are statistically significant (<0.05).

Table S3 Correlation matrix between psychomotor changes, fatigue, and appetite changes in 124,976 participants with online survey data [a]

**Symptoms**	psychomotor changes	fatigue	appetite changes
psychomotor changes	1		
fatigue	0.173	1	
appetite changes	0.215	0.333	1

a: Correlation coefficients were Phi coefficients.

Table S4 Interactions between biological age accelerations and age/sex in predicting incident depression/anxiety disorders at follow-up

I. Interaction test

Age	*p*-values of interaction [a]
KDM-BA acceleration	0.185
PhenoAge acceleration	0.214

Sex	*p*-values of interaction [a]
KDM-BA acceleration	0.052
PhenoAge acceleration	**0.01**

a: Model adjusted for age, sex, ethnic, BMI, smoking status, healthy alcohol intake, healthy physical activity, hypertension, diabetes, coronary heart disease, Townsend deprivation index, and the interaction terms of biological age accelerations with age/sex. The examination center was controlled for as a random effect. Two-sided statistical tests were conducted and no adjustments were made for multiple comparisons. Bolded p-values are statistically significant (<0.05).

II. Associations between PhenoAge acceleration with incident depression/anxiety disorders at follow-up by sex [a]

PhenoAge	Female			Male		
	N_{case}/N_{total}	Hazard ratio (95% CI)	*p*-value	N_{case}/N_{total}	Hazard ratio (95% CI)	*p*-value
Per one SD increase	10529 / 195774	1.092 (1.071 – 1.114)	**<0.0001**	5922 / 173971	1.134 (1.106 – 1.163)	**<0.0001**

a: Model adjusted for age, ethnic, BMI, smoking status, healthy alcohol intake, healthy physical activity, hypertension, diabetes, coronary heart disease, and Townsend deprivation index. The examination center was controlled for as a random effect. Two-sided statistical tests were conducted and no adjustments were made for multiple comparisons. Bolded p-values are statistically significant (<0.05).

Table S5 Associations of biological ages at baseline with incident depression/anxiety disorders at follow-up among individuals with >2 years of follow-up [a]

Biological age	Incident depression/anxiety disorders			Incident depression			Incident anxiety		
	N_{case}/N_{total}	**Hazard ratio (95% CI)**	***p*-value**	N_{case}/N_{total}	**Hazard ratio (95% CI)**	***p*-value**	N_{case}/N_{total}	**Hazard ratio (95% CI)**	***p*-value**
KDM-BA acceleration (Continuous)	16397 / 368311	1.054 (1.028 – 1.080)	**<0.0001**	10721 / 368311	1.066 (1.035 – 1.099)	**<0.0001**	8453 / 368311	1.034 (0.999 – 1.071)	0.06
KDM-BA acceleration (Quartiles)									
Q1	3701 / 92077	Ref		2227 / 92077	Ref		2091 / 92077	Ref	
Q2	3722 / 92078	1.099 (1.032 – 1.172)	**0.0034**	2361 / 92078	1.080 (0.999 – 1.185)	0.05	2013 / 92078	1.050 (0.956 – 1.153)	0.31
Q3	4127 / 92078	1.100 (1.043 – 1.161)	**0.0005**	2750 / 92078	1.110 (1.039 – 1.185)	**0.0019**	2084 / 92078	1.106 (1.011 – 1.209)	**0.028**
Q4	4847 / 92078	1.119 (1.047 – 1.195)	**0.0009**	3383 / 92078	1.160 (1.070 – 1.257)	**0.0003**	2265 / 92078	1.076 (0.997 – 1.161)	0.06
PhenoAge acceleration (Continuous)	16397 / 368311	1.107 (1.090 – 1.124)	**<0.0001**	10721 / 368311	1.141 (1.121 – 1.163)	**<0.0001**	8453 / 368311	1.059 (1.036 – 1.083)	**<0.0001**
PhenoAge acceleration (Quartiles)									
Q1	3701 / 92077	Ref		2227 / 92077	Ref		2091 / 92077	Ref	
Q2	3722 / 92078	1.030 (0.983 – 1.078)	0.21	2361 / 92078	1.066 (1.006 – 1.131)	**0.031**	2013 / 92078	1.009 (0.948 – 1.074)	0.77
Q3	4127 / 92078	1.131 (1.081 – 1.184)	**<0.0001**	2750 / 92078	1.210 (1.142 – 1.282)	**<0.0001**	2084 / 92078	1.058 (0.944 – 1.126)	0.08
Q4	4847 / 92078	1.272 (1.215 – 1.332)	**<0.0001**	3383 / 92078	1.392 (1.314 – 1.474)	**<0.0001**	2265 / 92078	1.140 (1.070 – 1.215)	**<0.0001**

a: Analyses were conducted in 368,311 participants free of depression/anxiety at baseline and with >2 years of follow-up; Model adjusted for age, sex, ethnic, BMI, smoking status, healthy alcohol intake, healthy physical activity, hypertension, diabetes, coronary heart disease, and Townsend deprivation index. The examination center was controlled for as a random effect. Two-sided statistical tests were conducted and no adjustments were made for multiple comparisons. Bolded p-values are statistically significant (<0.05).

Table S6 Incident diabetes, cardiovascular diseases, and cancers by incident depression/anxiety status among 369,745 participants free of depression/anxiety at baseline [a]

Incident depression/anxiety	Incident diabetes (%)	Incident cardiovascular diseases (%)	Incident cancers (%)
Yes (N = 16523)	956 (5.8%)	1923 (11.6%)	1835 (11.1%)
No (N = 353222)	10431 (2.9%)	20868 (5.9%)	27738 (7.9%)

a: Incident cases of diabetes/cardiovascular diseases/cancers in UK Biobank were obtained through regular linkages to multiple national databases based on corresponding ICD-10 codes. All *p*-values for the chi-square test between incident depression/anxiety cases and incident diabetes/cardiovascular diseases/cancers were <0.0001. Two-sided statistical tests were conducted and no adjustments were made for multiple comparisons.

Table S7 Associations of biological age accelerations at baseline with incident depression/anxiety disorders at follow-up by additionally controlling for whether individuals diagnosed with incident diabetes, cardiovascular diseases, or cancer during the follow-up

Predictors	Incident depression/anxiety disorders			Incident depression			Incident anxiety		
	N_{case}/N_{total}	Hazard ratio (95% CI)	*p*-value	N_{case}/N_{total}	Hazard ratio (95% CI)	*p*-value	N_{case}/N_{total}	Hazard ratio (95% CI)	*p*-value
KDM-BA acceleration	16523 / 369745	1.040 (1.015 – 1.066)	**0.0015**	11042 / 369745	1.054 (1.023 – 1.086)	**0.0006**	8472 / 369745	1.020 (1.000 – 1.056)	**0.049**
PhenoAge acceleration	16523 / 369745	1.095 (1.078 – 1.112)	**<0.0001**	11042 / 369745	1.129 (1.108 – 1.150)	**<0.0001**	8472 / 369745	1.046 (1.023 – 1.070)	**<0.0001**

a: Analysis was conducted in 369,745 baseline depression/anxiety-free participants. Model adjusted for age, sex, ethnic, BMI, smoking status, healthy alcohol intake, healthy physical activity, hypertension, diabetes, coronary heart disease, Townsend deprivation index, and the indicator whether individuals diagnosed with incident diabetes, cardiovascular diseases, or cancer during the follow-up. The examination center was controlled for as a random effect. Estimates were reported by per one SD increase in age accelerations. Two-sided statistical tests were conducted and no adjustments were made for multiple comparisons.

Table S8 Associations of biological age accelerations at baseline with incident depression/anxiety disorders at follow-up among individuals with available follow-up survey data by additionally controlling for childhood adversity

**Predictors**	**Incident depression/anxiety disorders**			**Incident depression**			**Incident anxiety**		
	**N_{case}/N_{total}**	**Hazard ratio (95% CI)**	***p*-value**	**N_{case}/N_{total}**	**Hazard ratio (95% CI)**	***p*-value**	**N_{case}/N_{total}**	**Hazard ratio (95% CI)**	***p*-value**
Model included KDM-BA acceleration and childhood adversity									
KDM-BA acceleration	8094 / 124976	1.058 (1.019 – 1.097)	**0.0029**	5284 / 124976	1.062 (1.016 – 1.111)	**0.0083**	4439 / 124976	1.034 (1.001 – 1.064)	**0.043**
Childhood adversity	8094 / 124976	1.376 (1.354 – 1.399)	**<0.0001**	5284 / 124976	1.418 (1.391 – 1.446)	**<0.0001**	4439 / 124976	1.369 (1.339 – 1.399)	**<0.0001**
Model included PhenoAge acceleration and childhood adversity									
PhenoAge acceleration	8094 / 124976	1.063 (1.040 – 1.087)	**<0.0001**	5284 / 124976	1.096 (1.067 – 1.126)	**<0.0001**	4439 / 124976	1.021 (1.000 – 1.043)	**0.049**
Childhood adversity	8094 / 124976	1.376 (1.354 – 1.398)	**<0.0001**	5284 / 124976	1.417 (1.390 – 1.445)	**<0.0001**	4439 / 124976	1.368 (1.339 – 1.399)	**<0.0001**

a: Analysis was conducted in 124,976 baseline depression/anxiety-free participants with available follow-up survey data. Model adjusted for age, sex, ethnic, BMI, smoking status, healthy alcohol intake, healthy physical activity, hypertension, diabetes, coronary heart disease, Townsend deprivation index, and the numbers of childhood adversities. The examination center was controlled for as a random effect. Estimates were reported by per one SD increase in age accelerations and one adversity increase in the numbers of childhood adversities. Two-sided statistical tests were conducted and no adjustments were made for multiple comparisons.

Table S9 Associations of the odds of depression/anxiety disorders and PHQ-4 score with polygenic risk scores at baseline [a]

Trait	Coefficients	SE	R-squares (variance explained)	*p*-value
Prevalent depression	0.4139	0.0068	0.0958	**<0.0001**
Prevalent anxiety	0.0367	0.0053	0.0771	**<0.0001**
Prevalent depression/anxiety	0.3275	0.0049	0.0990	**<0.0001**
PHQ-4 score	0.2915	0.0031	0.0967	**<0.0001**

a: Associations of depression, anxiety, and either disorder at baseline with polygenic risk scores were tested by logistic regression models; association of PHQ-4 score with polygenic risk score of depression/anxiety disorders were tested by linear regression models. Models were adjusted for age, sex, ethnic, BMI, smoking status, healthy alcohol intake, healthy physical activity, hypertension, diabetes, coronary heart disease, and Townsend deprivation index. Two-sided statistical tests were conducted and no adjustments were made for multiple comparisons.

Table S10 Associations of polygenic risk score with odds of depression/anxiety disorders at baseline, incident depression/anxiety disorders at follow-up, and the accelerations of two biological ages [a]

I.

**Polygenic risk score**	**Odds of depression/anxiety disorders at baseline**			**Incident depression/anxiety disorders at follow-up**		
	N_{case}/N_{total}	Odds ratio (95% CI)	*p*-value	N_{case}/N_{total}	Hazard ratio (95% CI)	*p*-value
Per one SD increase	54554 / 424299	1.386 (1.373 – 1.400)	**<0.0001**	16523 / 369745	1.495 (1.473 – 1.518)	**<0.0001**

II.

**Polygenic risk score**	**KDM-BA acceleration**		**PhenoAge acceleration**	
	Coefficients (SE)	*p*-value	Coefficients (SE)	*p*-value
Per one SD increase	0.0017 (0.0011)	0.10	0.0046 (0.0024)	0.06

a: Model adjusted for age, sex, ethnic, BMI, smoking status, healthy alcohol intake, healthy physical activity, hypertension, diabetes, coronary heart disease, and Townsend deprivation index. The examination center was controlled for as a random effect. Two-sided statistical tests were conducted and no adjustments were made for multiple comparisons. Bolded p-values are statistically significant (<0.05).

Table S11 Mutual associations of biological age accelerations and polygenic risk score with odds of depression/anxiety disorders at baseline and incident depression/anxiety disorders at follow-up [a]

**Odds of depression/anxiety disorders at baseline**	**OR (95% CI)**				***p*-values of interaction** [c]
	**Biological age accelerations**	***p*-values**	**Polygenic risk score**	***p*-values**	
KDM-BA acceleration	1.123 (1.108 – 1.139)	**<0.0001**	1.386 (1.373 – 1.400)	**<0.0001**	0.14
PhenoAge acceleration	1.152 (1.141 – 1.163)	**<0.0001**	1.386 (1.373 – 1.400)	**<0.0001**	**0.047**

**Incident depression/anxiety disorders at follow-up**	**HR (95% CI)**				***p*-values of interaction** [c]
	**Biological age accelerations**	***p*-values**	**Polygenic risk score**	***p*-values**	
**KDM-BA acceleration**	1.067 (1.041 – 1.093)	**<0.0001**	1.496 (1.473 – 1.519)	**<0.0001**	**0.048**
**PhenoAge acceleration**	1.116 (1.099 – 1.134)	**<0.0001**	1.496 (1.473 – 1.519)	**<0.0001**	0.75

a: Model adjusted for age, sex, ethnic, BMI, smoking status, healthy alcohol intake, healthy physical activity, hypertension, diabetes, coronary heart disease, and Townsend deprivation index. The examination center was controlled for as a random effect. Interaction models adjusted for the interaction terms of biological age accelerations and genetic risk score additionally. Estimates were demonstrated per one SD increase in the biological age accelerations and per one SD increase in the polygenic risk score of depression/anxiety disorders. Two-sided statistical tests were conducted and no adjustments were made for multiple comparisons. Bolded p-values are statistically significant (<0.05).

b: Estimates were from models adjusted for both factors without the interaction terms.

c: *p*-values of interaction terms in interaction models.

Table S12 Joint associations of polygenic risk score and biological age accelerations with the odds of depression/anxiety disorders at baseline and risks of incident depression/anxiety disorders during follow-up [a]

I. Odds of depression/anxiety disorders

**Categories**	**OR (95%CI)**	***p*-values**
**PRS & KDM-BA acceleration**		
Low and Q1	Ref.	
Low and Q2	1.186 (1.132 - 1.244)	**<0.0001**
Low and Q3	1.244 (1.183 - 1.309)	**<0.0001**
Low and Q4	1.357 (1.290 - 1.427)	**<0.0001**
High and Q1	1.774 (1.698 - 1.853)	**<0.0001**
High and Q2	1.951 (1.867 - 2.039)	**<0.0001**
High and Q3	2.082 (1.985 - 2.184)	**<0.0001**
High and Q4	2.324 (2.215 - 2.439)	**<0.0001**
**PRS & PhenoAge acceleration**		
Low and Q1	Ref.	
Low and Q2	1.042 (0.996 - 1.089)	0.07
Low and Q3	1.106 (1.058 - 1.155)	**<0.0001**
Low and Q4	1.319 (1.263 - 1.376)	**<0.0001**
High and Q1	1.682 (1.614 - 1.753)	**<0.0001**
High and Q2	1.720 (1.651 - 1.792)	**<0.0001**
High and Q3	1.881 (1.806 - 1.959)	**<0.0001**
High and Q4	2.287 (2.197 - 2.380)	**<0.0001**

II. Risks of incident depression/anxiety disorders

Categories	HR (95%CI)	*p*-values
**PRS & KDM-BA acceleration**		
Low and Q1	Ref.	
Low and Q2	1.136 (1.041 - 1.240)	**0.0007**
Low and Q3	1.126 (1.027 - 1.234)	**0.0020**
Low and Q4	1.180 (1.075 - 1.295)	**<0.0001**
High and Q1	2.071 (1.914 - 2.240)	**<0.0001**
High and Q2	2.181 (2.016 - 2.360)	**<0.0001**
High and Q3	2.163 (1.987 - 2.356)	**<0.0001**
High and Q4	2.201 (2.018 - 2.401)	**<0.0001**
**PRS & PhenoAge acceleration**		
Low and Q1	Ref.	
Low and Q2	1.047 (0.968 - 1.132)	0.25
Low and Q3	1.131 (1.047 - 1.222)	**0.0019**
Low and Q4	1.324 (1.227 - 1.428)	**<0.0001**
High and Q1	1.980 (1.850 - 2.119)	**<0.0001**
High and Q2	1.995 (1.863 - 2.137)	**<0.0001**
High and Q3	2.221 (2.076 - 2.377)	**<0.0001**
High and Q4	2.483 (2.321 - 2.656)	**<0.0001**

a: Model adjusted for age, sex, ethnic, BMI, smoking status, healthy alcohol intake, healthy physical activity, hypertension, diabetes, coronary heart disease, and Townsend deprivation index. The examination center was controlled for as a random effect. Two-sided statistical tests were conducted and no adjustments were made for multiple comparisons. Bolded p-values are statistically significant (<0.05).

Table S13 Full names and data field IDs of variables for the construction of biological ages

Labels in the current study	Full name in UK Biobank data dictionary	Field ID
FEV_1 (L)	Forced expiratory volume in 1-second (FEV_1)	3063
SBP (mm Hg)	Systolic blood pressure, automated reading	4080
Total Cholesterol (mg/dL)	Cholesterol	30690
Glycated hemoglobin (%)	Glycated haemoglobin (HbA1c)	30750
Blood urea nitrogen (mg/dL)	Urea	30670
Lymphocyte (%)	Lymphocyte percentage	30180
Mean cell volume (fL)	Mean sphered cell volume	30270
Serum glucose (mg/dL)	Glucose	30740
Red cell distribution width (%)	Red blood cell (erythrocyte) distribution width	30070
White blood cell count (1000 cells/uL)	White blood cell (leukocyte) count	30000
Albumin (g/dL)	Albumin	30600
Creatinine (mg/dL)	Creatinine	30700
C-reactive protein (mg/dL)	C-reactive protein	30710
Alkaline phosphatase (U/L)	Alkaline phosphatase	30610

Table S14 Best-fitting parameters of the polygenetic risk scores for depression, anxiety, and both disorders

**Outcomes**	**Full R-squares**	**Numbers of selected SNPs**
Depression	0.0556607	152817
Anxiety	0.0123399	14193
Both disorders	0.0489046	152587

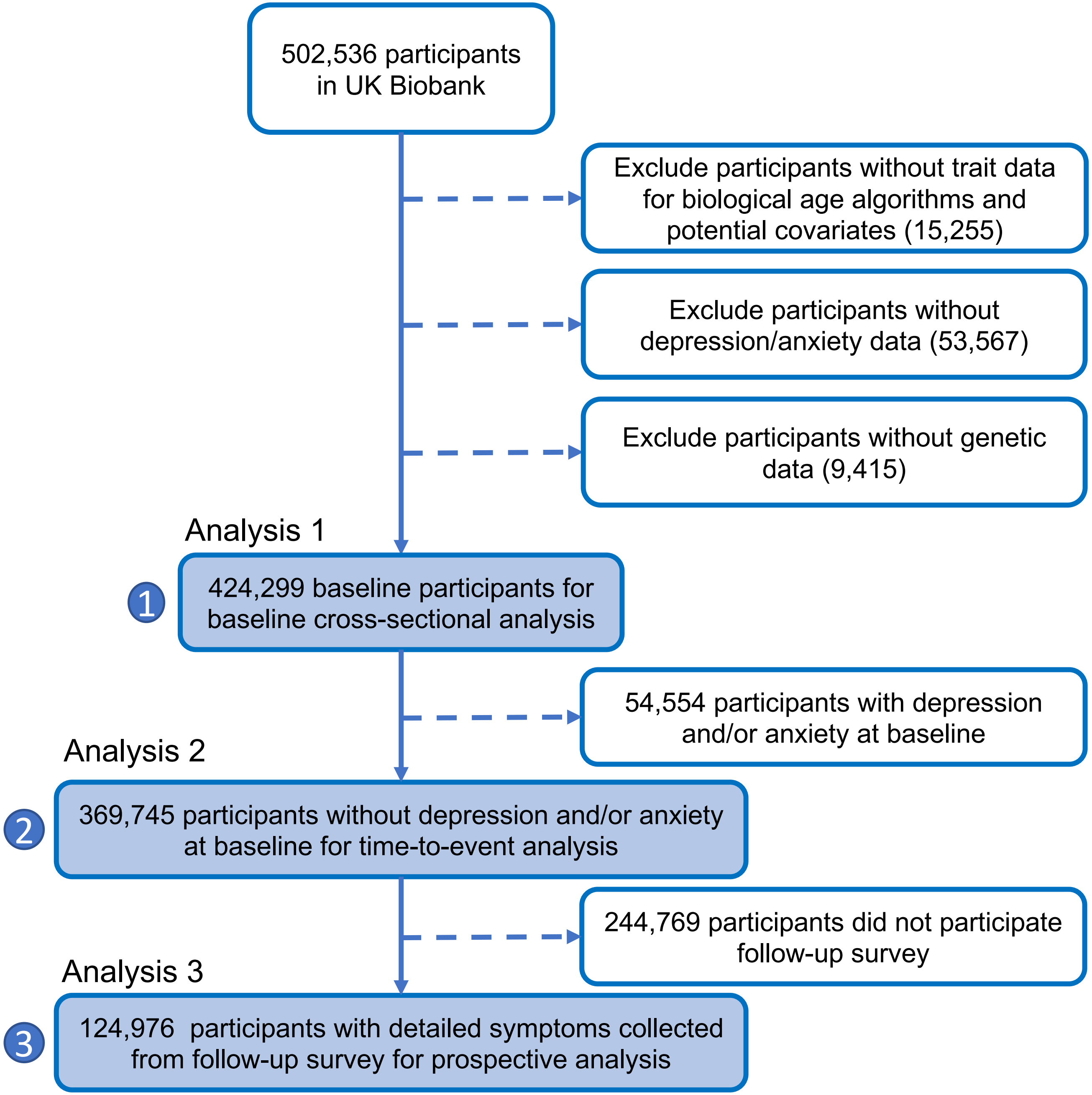

Figure S1 Flowchart for the selection of study participants and three subgroups for analysis

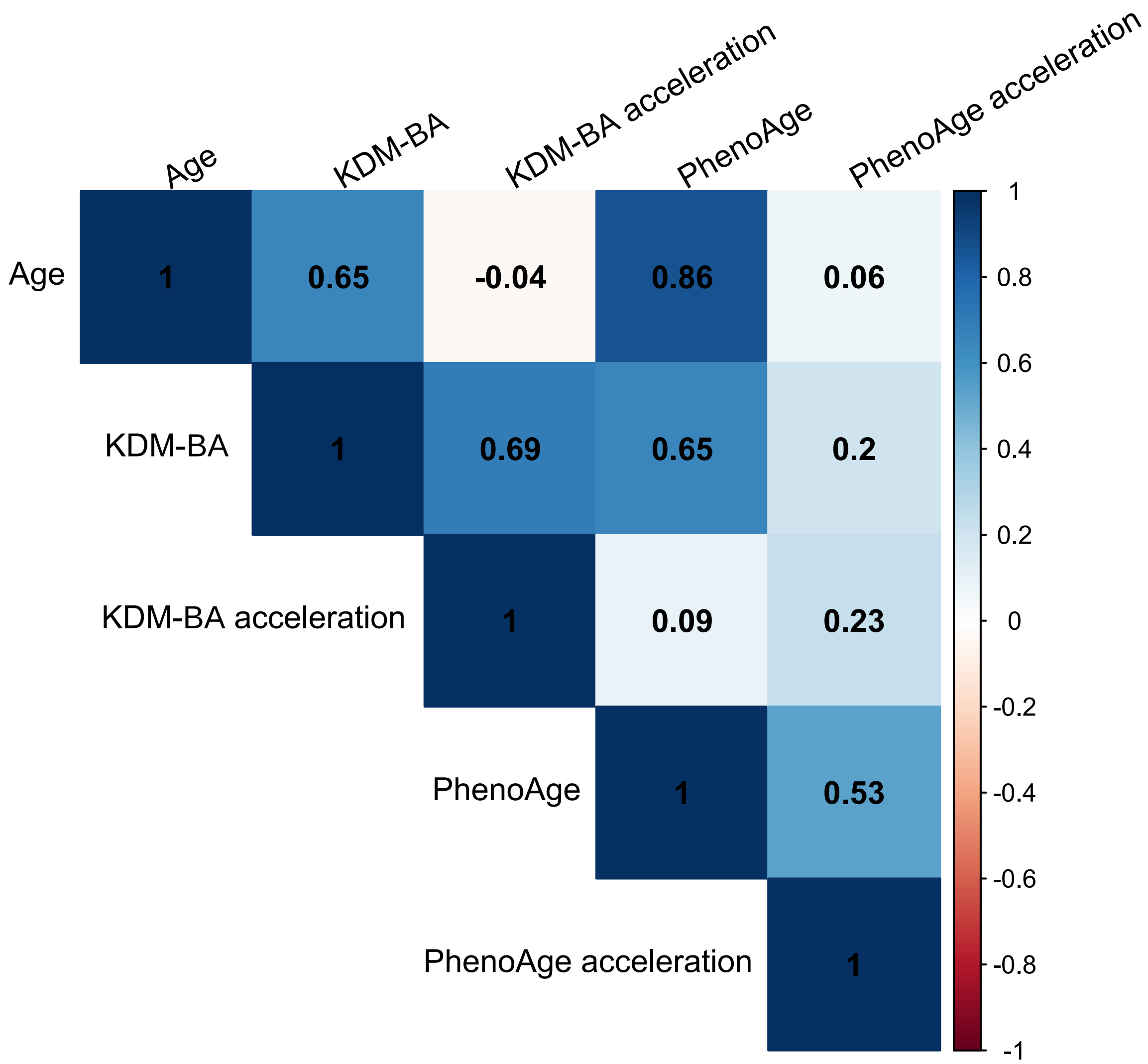

Figure S2 Correlation matrix of chronological age, biological ages, and age accelerations (Pearson correlation)

Source data are provided as a Source Data file.
