# Supplementary material for: Accelerated biological aging and risk of depression and anxiety: evidence from 424,299 UK Biobank participants
Source: Nat Commun. 2023 Apr 20;14:2277. doi: 10.1038/s41467-023-38013-7 (PMC10119095; doi:10.1038/s41467-023-38013-7)
Supplement: Supplementary file 2 — Reporting Summary [file 41467_2023_38013_MOESM2_ESM.pdf]

nature portfolio

Corresponding author(s): Dr. Xu Gao; Dr. Daniel W. Belsky

Last updated by author(s): Mar 29, 2023

Reporting Summary

Nature Portfolio wishes to improve the reproducibility of the work that we publish. This form provides structure for consistency and transparency in reporting. For further information on Nature Portfolio policies, see our Editorial Policies and the Editorial Policy Checklist.

Statistics

For all statistical analyses, confirm that the following items are present in the figure legend, table legend, main text, or Methods section.

n/a	Confirmed	
☐	☒	The exact sample size (*n*) for each experimental group/condition, given as a discrete number and unit of measurement
☐	☒	A statement on whether measurements were taken from distinct samples or whether the same sample was measured repeatedly
☐	☒	The statistical test(s) used AND whether they are one- or two-sided<br>*Only common tests should be described solely by name; describe more complex techniques in the Methods section.*
☐	☒	A description of all covariates tested
☐	☒	A description of any assumptions or corrections, such as tests of normality and adjustment for multiple comparisons
☐	☒	A full description of the statistical parameters including central tendency (e.g. means) or other basic estimates (e.g. regression coefficient) AND variation (e.g. standard deviation) or associated estimates of uncertainty (e.g. confidence intervals)
☐	☒	For null hypothesis testing, the test statistic (e.g. *F*, *t*, *r*) with confidence intervals, effect sizes, degrees of freedom and *P* value noted<br>*Give P values as exact values whenever suitable.*
☒	☐	For Bayesian analysis, information on the choice of priors and Markov chain Monte Carlo settings
☐	☒	For hierarchical and complex designs, identification of the appropriate level for tests and full reporting of outcomes
☐	☒	Estimates of effect sizes (e.g. Cohen's *d*, Pearson's *r*), indicating how they were calculated

Our web collection on statistics for biologists contains articles on many of the points above.

Software and code

Policy information about availability of computer code

Data collection	The data collection of this study used questionnaires and clinical lab tests. The collected information was imported into SAS ver 9.4 to save as SAS datasets.
Data analysis	SAS version 9.4 TS1M7 and R version 4.0 was used to perform data analyses. PRS is constructed using the METAL software and PRSice-2 v2.3.5 software. The codes are available upon request from Dr. Xu Gao.

For manuscripts utilizing custom algorithms or software that are central to the research but not yet described in published literature, software must be made available to editors and reviewers. We strongly encourage code deposition in a community repository (e.g. GitHub). See the Nature Portfolio guidelines for submitting code & software for further information.

Data

Policy information about availability of data

All manuscripts must include a data availability statement. This statement should provide the following information, where applicable:

- Accession codes, unique identifiers, or web links for publicly available datasets
- A description of any restrictions on data availability
- For clinical datasets or third party data, please ensure that the statement adheres to our policy

Data are available in a public, open access repository. This research has been conducted using the UK Biobank Resource under Application Number 44430. The UK Biobank data are available on application to the UK Biobank (www.ukbiobank.ac.uk/) with access fees.

Human research participants

Policy information about studies involving human research participants and Sex and Gender in Research.

Reporting on sex and gender	The reported sex of participants was accessed through UK Biobank data-field 31, and included as a covariate in the statistical analyses. Sex proportion was reported and one of the sensitivity analyses was conducted by sex. Gender was not considered in this study.
Population characteristics	The participants were mostly White (>95%), 46.2% were male, aged 57±8 year-old, with substantial prevalence of hypertension (54%) and some prevalence of coronary heart disease (3-5%) and diabetes (3-5%). In the first group, baseline PHQ-4 scores averaged 1.58±2.08. Based on PHQ-4 scores and hospital records, 13% met criteria for having the symptoms of depression (6%) and anxiety (10%). In the second group, who were free of depression and anxiety at baseline, depression and anxiety were incident in 4.47% (Ndepression=11,402, Nanxiety=8,472, and Nboth=2,991) over a median of 8.7 years of follow-up. In the third group, who were free of depression/anxiety at baseline and who participated in the follow-up survey, depression and anxiety were incident in 4.9% (Ndepression=4,230, Nanxiety=3,347, and Nboth=1,501) at online follow-up.
Recruitment	UK Biobank is an ongoing prospective study with 502,536 participants recruited in 2006–2010 at the age of 37–73 years (baseline survey) with multiple follow-ups. At baseline, participants were asked to provide information on their lifestyle and health, and their biological samples were collected. During 2016-2017, the mental health status of ~1/3 participants was obtained via an online survey (i.e., follow-up survey).
Ethics oversight	UK Biobank research has received approval from the North West Multicenter Research Ethical Committee. Written informed consents were provided by all participants.

Note that full information on the approval of the study protocol must also be provided in the manuscript.

Field-specific reporting

Please select the one below that is the best fit for your research. If you are not sure, read the appropriate sections before making your selection.

☒ Life sciences ☐ Behavioural & social sciences ☐ Ecological, evolutionary & environmental sciences

For a reference copy of the document with all sections, see nature.com/documents/nr-reporting-summary-flat.pdf

Life sciences study design

All studies must disclose on these points even when the disclosure is negative.

Sample size	UK Biobank is an ongoing prospective study with 502,536 participants recruited in 2006–2010 at the age of 37–73 years (baseline survey) with multiple follow-ups. At baseline, participants were asked to provide information on their lifestyle and health, and their biological samples were collected. During 2016-2017, the mental health status of ~1/3 participants was obtained via an online survey (i.e., follow-up survey). We chose this sample size because of the data availability.<br><br>In this study, we included 424,299 participants with available data on mental health status, measures of traits for included in biological age algorithms, potential covariates, and genetic variants at baseline. According to the rule-of-thumb estimation, at least ten events are required per variable (including dummy variables) in the model (Riley et al. BMJ. 2020). In the fully adjusted models, we included a total of 18 variables (including dummy variables) in the Cox regression models. Thus, at least 180 events for each outcome were required. The events for depression/anxiety, depression, and anxiety were 54,554, 26,424, and 43,544, respectively. Therefore, the sample size of this study should be sufficient.
Data exclusions	In this study, we included 424,299 participants with available data on mental health status, measures of traits for included in biological age algorithms, potential covariates, and genetic variants at baseline. After excluding 54,554 participants with depression/anxiety at baseline, we conducted another two analyses in the remaining 369,745 participants who were free of depression or anxiety at baseline. The total 369,745 participants who were free of depression or anxiety at baseline was employed to assess the association between biological aging and the incidence of depression and/or anxiety. A subset of 124,976 of the 369,745 participants reported their mental health status at the follow-up survey were used to evaluate the prospective associations between baseline biological aging and the syndromes of depression or anxiety in between.
Replication	This is a population-based epidemiological cohort study, the findings of which may need to be validated by further clinical trials and/or larger cohorts.
Randomization	This is a population-based epidemiological cohort study. An observational study where the researcher observes the events and does not control them. It does not require randomization to allocate patients and controls. The covariates were controlled using the regression models which included all potential covariates.
Blinding	This is a population-based epidemiological cohort study which does not perform blinding to give treatments or placebo as randomized controlled trials.

Reporting for specific materials, systems and methods

We require information from authors about some types of materials, experimental systems and methods used in many studies. Here, indicate whether each material, system or method listed is relevant to your study. If you are not sure if a list item applies to your research, read the appropriate section before selecting a response.

Materials & experimental systems

n/a	Involved in the study
☒	☐ Antibodies
☒	☐ Eukaryotic cell lines
☒	☐ Palaeontology and archaeology
☒	☐ Animals and other organisms
☒	☐ Clinical data
☒	☐ Dual use research of concern

Methods

n/a	Involved in the study
☒	☐ ChIP-seq
☒	☐ Flow cytometry
☒	☐ MRI-based neuroimaging
